# Releasable, Immune‐Instructive, Bioinspired Multilayer Coating Resists Implant‐Induced Fibrosis while Accelerating Tissue Repair

**DOI:** 10.1002/adhm.202302611

**Published:** 2023-12-19

**Authors:** Riki Toita, Masahiro Kitamura, Akira Tsuchiya, Jeong‐Hun Kang, Shinjiro Kasahara

**Affiliations:** ^1^ Biomedical Research Institute National Institute of Advanced Industrial Science and Technology (AIST) 1‐8‐31 Midorigaoka Ikeda Osaka 563‐8577 Japan; ^2^ AIST‐Osaka University Advanced Photonics and Biosensing Open Innovation Laboratory AIST 2‐1 Yamadaoka Suita Osaka 565‐0871 Japan; ^3^ Niterra Co., Ltd. 2808 Iwasaki Komaki Aichi 485–8510 Japan; ^4^ NGK Spark Plug‐AIST Healthcare Materials Cooperative Research Laboratory 2266–98 Anagahora Shimoshidami, Moriyama‐ku Nagoya Aichi 463–8560 Japan; ^5^ Department of Biomaterials Faculty of Dental Science Kyushu University 3‐1‐1 Maidashi Higashi‐ku Fukuoka 812–8582 Japan; ^6^ Division of Biopharmaceutics and Pharmacokinetics National Cerebral and Cardiovascular Center Research Institute 6‐1 Shinmachi, Kishibe Suita Osaka 564–8565 Japan

**Keywords:** fibrosis, foreign body reaction, layer‐by‐layer, macrophage, phosphatidylserine liposome

## Abstract

Implantable biomaterials trigger foreign body reactions (FBRs), which reduces the functional life of medical devices and prevents effective tissue regeneration. Although existing therapeutic approaches can circumvent collagen‐rich fibrotic encapsulation secondary to FBRs, they disrupt native tissue repair. Herein, a new surface engineering strategy in which an apoptotic‐mimetic, immunomodulatory, phosphatidylserine liposome (PSL) is released from an implant coating to induce the formation of a macrophage phenotype that mitigates FBRs and improves tissue healing is described. PSL‐multilayers constructed on implant surfaces via the layer‐by‐layer method release PSLs over a 1‐month period. In rat muscles, poly(etheretherketone) (PEEK), a nondegradable polymer implant model, induces FBRs with dense fibrotic scarring under an aberrant cellular profile that recruits high levels of inflammatory infiltrates, foreign body giant cells (FBGCs), scar‐forming myofibroblasts, and inflammatory M1‐like macrophages but negligible amounts of anti‐inflammatory M2‐like phenotypes. However, the PSL‐multilayer coating markedly diminishes these detrimental signatures by shifting the macrophage phenotype. Unlike other therapeutics, PSL‐multilayered coatings also stimulate muscle regeneration. This study demonstrates that PSL‐multilayered coatings are effective in eliminating FBRs and promoting regeneration, hence offering potent and broad applications for implantable biomaterials.

## Graphical abstract

Implanted materials face encapsulation and eventual rejection in a dense, fibrotic scar because they inevitably trigger foreign body reactions (FBRs) of the immune system. A multilayered coating comprising phosphatidylserine liposomes (PSLs), artificial apoptotic mimetics, mitigates FBRs, chronic inflammation, multinucleated giant cell formation, and eventual fibrotic scarring associated with implanted devices while promoting tissue repair through M1‐to‐M2 macrophage transition.

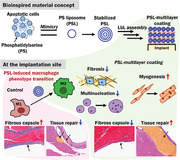

## 1 Introduction

Implantable medical devices are an integral part of modern medicine, performing diagnostic, therapeutic, and regenerative functions in various forms, such as blood glucose sensors, pacemakers, vascular stents, drug delivery platforms, neural interfaces, orthopedic/dental implants, scaffolds for tissue regeneration, and cell encapsulation/transplantation.^[^ ^1^, ^2^, ^3^, ^4^, ^5^ ^]^ However, they elicit foreign body reactions (FBRs) of the immune system, and thus they are encapsulated and eventually rejected in a dense fibrotic scar.^[^ ^3^, ^4^, ^5^, ^6^ ^]^ This results in painful tissue degeneration, inhibited molecular transport, reduced device longevity and functionality, impaired tissue–device integration, delayed tissue healing, and, in some cases, surgical revision. Growing evidence suggests that macrophages are key immune cells that drive a wide range of FBRs and wound healing processes.^[^ ^3^, ^4^, ^5^, ^6^, ^7^ ^]^ Macrophages progressively accumulate adjacent or adhere to engrafted devices, triggering acute inflammatory responses. Thereafter, they participate in the establishment of a chronic inflammatory niche, wherein some macrophages fuse into foreign body giant cells (FBGCs), which cooperatively amplify immune responses. Several factors secreted from macrophages and FBGCs activate the transformation of fibroblasts into myofibroblasts, leading to the formation of a collagenous fibrotic capsule that envelops the device and prevents full tissue healing.

Chronic inflammation is the primary culprit behind implant‐mediated fibrosis as well as a variety of fibrotic diseases. Attempts to minimize FBRs often involve anti‐proliferative or immunosuppressive agents, such as steroids, non‐steroidal anti‐inflammatory drugs (NSAIDs), and anti‐inflammatory cytokines.^[^ ^3^, ^4^, ^5^ ^]^ They attenuate acute inflammation and FBRs. However, sustained systemic usage is needed to reduce FBRs, which leads to systemic adverse complications, such as intestinal ulcers, hepatic, cardiac, or renal toxicities, and microbial dysbiosis.^[^ ^8^, ^9^, ^10^ ^]^ Localized drug delivery systems, specifically drug‐eluting composites or coating and polymeric microspheres, have the potential to solve this safety concern, although they suffer from burst release and cannot provide long‐term controlled release.^[^ ^11^, ^12^, ^13^, ^14^ ^]^ Another potential drawback includes the heightened inflammation reaction caused by synthetic polymers, which are necessary to formulate controlled release systems.^[^ ^14^ ^]^ A synthetic polymer‐free coating based on dimeric dexamethasone has recently been demonstrated to mitigate FBRs for 45 days, corresponding to the sustained release profile.^[^ ^15^ ^]^ Nevertheless, even with advances in controlled release systems, current therapeutic agents aimed at preventing FBRs disturb the original healing process in various organs.^[^ ^16^, ^17^, ^18^, ^19^, ^20^, ^21^, ^22^, ^23^, ^24^ ^]^ Overall, a therapeutic strategy that can ameliorate FBRs while enhancing tissue regeneration remains challenging.

The biological aspects involved in macrophage functions during inflammation resolution and tissue repair inspired us to conceptualize a new therapeutic engineering strategy. Macrophage‐mediated removal of apoptotic cells is the decisive step in dampening inflammation and promoting damaged‐tissues regeneration, thereby preventing chronic inflammation and maintaining tissue homeostasis.^[^ ^25^ ^]^ This is mediated by macrophage recognition of phosphatidylserine (PS), an anionic phospholipid found on the outer leaflet of apoptotic cell membranes.^[^ ^26^ ^]^ The PS acts as an “eat‐me” signal that promotes macrophage phagocytosis and transformation from the inflammatory (M1 type) to anti‐inflammatory/healing (M2 type) phenotype. PS liposomes (PSLs) are artificial apoptotic mimetics that serve as unique immunosuppressive agents with a high affinity for macrophages and the M1‐to‐M2 macrophage transition.^[^ ^27^, ^28^, ^29^ ^]^ Our recent studies have demonstrated the advantages of PSLs in treating chronic inflammatory diseases, such as suppression of fibrosis and facilitation of chronic wound repair.^[^ ^27^, ^28^, ^29^, ^30^, ^31^ ^]^ While PSLs induce local macrophage polarization in target tissues, the systemic cytokine profile is left intact, suggesting low risks for systemic side effects.^[^ ^31^ ^]^

In this proof‐of‐concept report, we prepared PSL‐multilayer coated implants, which were applied to rat muscles to examine FBRs, fibrotic scarring, and muscle healing along with macrophage phenotypes. Muscle implantation models allow assessment of both FBR and tissue healing, unlike subcutaneous implantation models.^[^ ^32^ ^]^ Poly(etheretherketone) (PEEK) was selected to model a clinically acceptable, non‐biodegradable polymer that has no toxicity but triggers a relatively strong inflammatory response and FBRs.^[^ ^33^, ^34^, ^35^, ^36^ ^]^ We propose that the PSL‐multilayer coating represents a simple and versatile technology to limit chronic inflammation and fibrotic scarring associated with implanted devices as well as to promote tissue healing.

## 2 Results

### 2.1 In Vitro Characterization of PSLs

**Figure 1:**
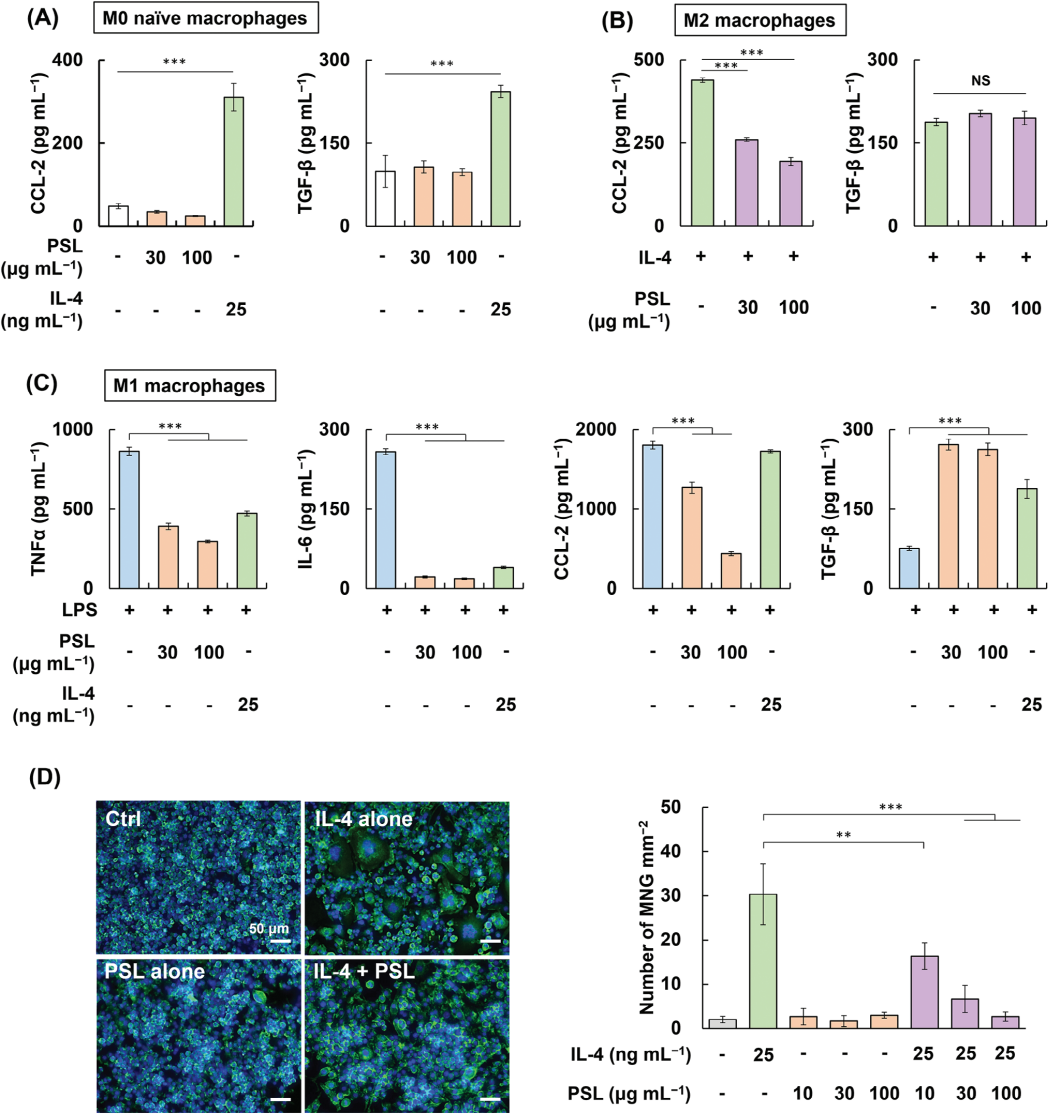
Biological characterization of phosphatidylserine liposome (PSL). Cytokine profiles of A) M0 (without stimulation), B) M2, and C) M1 macrophages at 24 h after treating with PSL plus PBS, 50 ng mL^−1^ IL‐4, and 100 ng mL^−1^ lipopolysaccharide (LPS), respectively (*n* = 3 or 4). For M0 and M1 macrophages, IL‐4 was used as the positive control. The concentrations of TNFα and IL‐6 in A) and B) were below the detection limit (data not shown). D) Formation of multinucleated giant cell (MNG) of macrophages six days after culturing in the presence or absence of IL‐4 and/or PSL (*n* = 4). Data are presented as mean ± SD. One‐way ANOVA with Tukey's multiple comparison tests were used. **p < 0.01; ***p < 0.001; NS, not significant.

The effect of PSL treatment on macrophage secretions was investigated using mouse macrophages with different phenotypes, such as naïve M0, lipopolysaccharide (LPS)‐primed inflammatory M1, and IL‐4‐activated anti‐inflammatory M2 macrophages. In M0 macrophages, IL‐4 treatment increased the levels of a typical M2 cytokine TGF‐β and an inflammatory chemokine CCL‐2 (**Figure** **1**A).^[^ ^7^, ^37^ ^]^ However, PSLs did not stimulate the production of TGF‐β and CCL‐2. Neither PSLs nor IL‐4 increased inflammatory TNFα and IL‐6 (data not shown). In M2 macrophages, PSLs significantly reduced IL‐4‐mediated CCL‐2 expression but did not alter the TGF‐β level (Figure 1B). M1 macrophages produced higher level of inflammatory TNFα, IL‐6, and CCL‐2 than M0 macrophages (Figure 1C). However, both PSLs and IL‐4 treatment decreased the TNFα and IL‐6 levels while increasing the TGF‐β levels. Furthermore, PSLs inhibited CCL‐2 secretion from M1 macrophages, but IL‐4 failed to reduce the CCL‐2 production because it is an inducer of CCL‐2, as observed in the M0 macrophage experiment (Figure 1A). Decreased TNFα and IL‐6 secretions were also found from PSLs‐treated human M1 macrophages (Figure S1, Supporting Information). These results demonstrated that PSLs specifically reacted with M1 inflammatory macrophages rather than M0 naïve macrophages and induced the M1‐to‐M2‐like macrophage polarization, unlike IL‐4, which polarized both M0 and M1 phenotypes into the M2 phenotype.

Furthermore, IL‐4 (mainly produced by mast cells and Th2 cells) promotes macrophage fusion and FBGC generation.^[^ ^3^, ^4^, ^5^, ^6^, ^38^ ^]^ The cell study consistently demonstrated that IL‐4 treatment of macrophages induced multinucleated giant cell formation, whereas PSL treatment did not (Figure 1D). Intriguingly, PSLs inhibited IL‐4‐mediated multinucleated giant cell formation in a dose‐dependent manner.

When PSLs (labeled with fluorescein lipids) were added to RAW 264.7 macrophages and C2C12 myoblasts for a comparison of cellular uptake, PSLs displayed a 6‐ to 10‐fold higher affinity for macrophages than for myoblasts (Figure S2, Supporting Information). PSLs exerted no detectable cytotoxic effects on these cells (Figure S3, Supporting Information). In the myotube atrophy assay, dexamethasone (positive control) markedly reduced the width of C2C12 myotubes,^[^ ^20^ ^]^ whereas no change was observed in the PSL group (Figure S4, Supporting Information). Myogenic differentiation of C2C12 cells remained intact even with PSLs (Figure S5, Supporting Information).

### 2.2 Impact of Macrophage Secretions on Fibroblast and Myoblast Responses

The effects of macrophage secretions on fibroblasts and myoblasts were studied using the conditioned medium (CM) from differentially activated macrophages. The CM used were “M0‐CM” from M0 macrophages, “M1‐CM” from M1 macrophages primed with LPS, and “M2‐CM” and “M1>M2‐CM” from M2‐like macrophages stimulated with PSL and LPS plus PSL, respectively. Note that M1>M2‐CM was obtained from M2‐like macrophages that were shifted from LPS‐primed M1 macrophages by PSL stimulation. Normal media (NM) without CM was used as a control.

**Figure 2:**
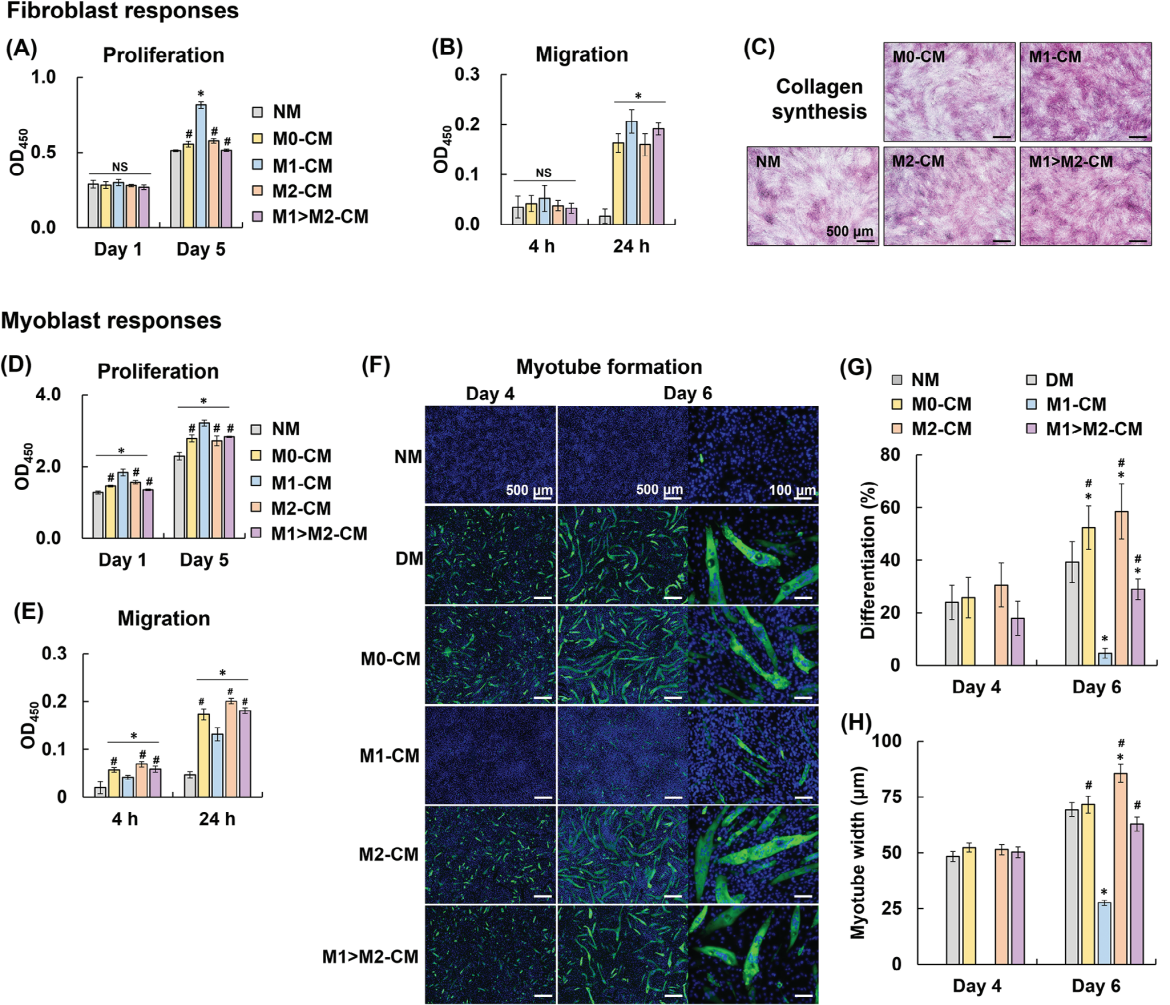
Cellular response in macrophage conditioned medium (CM). A) Proliferation (*n* = 5), B) migration (*n* = 3), and C) collagen synthesis of NIH/3T3 cells cultured in normal medium (NM) or macrophage CM. M0‐CM, M1‐CM, M2‐CM, and M1>M2‐CM were obtained from RAW 264.7 macrophages treated with PBS, lipopolysaccharide (LPS), phosphatidylserine liposome (PSL), and LPS plus PSL, respectively. Collagen is stained with Sirius Red. D) Proliferation (*n* = 5), E) migration (*n* = 3), and F−H) myotube formation (myogenic differentiation) of C2C12 cells. Myosin heavy chain, a marker of myotubes, is immunostained in green and nuclei are stained in blue. G) Level of myogenic differentiation (number of nuclei in the myotubes over total number of nuclei) (*n* = 8) and H) myotube width (*n* = 100−120). Data are presented as mean ± SD. One‐way ANOVA with Tukey's multiple comparison tests were used. NS, not significant; *p < 0.05 (versus NM or DM); # *p* < 0.05 (versus LPS‐CM).

Fibroblasts are responsible for fibrous capsule formation during FBRs, and thus the effects of macrophage secretions on NIH/3T3 fibroblast proliferation and migration as well as collagen synthesis were examined. NIH/3T3 fibroblast proliferation was dramatically greater in the M1‐CM group than in the other M0 and M2 CM groups (**Figure** **2**A). Cellular migration was enhanced in all CM groups compared with that in the NM group, whereas no difference was observed among the CM groups (Figure 2B). Collagen synthesis was augmented by the M1‐CM but was negatively affected by the CM from M0 and M2‐like macrophages (Figure 2C).

C2C12 cell proliferation (Figure 2D) and migration (Figure 2E) were enhanced in all the macrophage CM groups compared with those in the NM group. M1‐CM increased proliferation but decreased migration relative to the other CM. CM from M0 and M2 macrophages had similar effects on proliferation and migration. Myotube formation was observed four and six days after culturing in myogenic differentiation medium (DM), the positive control group (Figure 2F). M2‐CM further elevated myogenic differentiation (Figure 2G) and myotube width (Figure 2H) on day six. However, M1‐CM drastically suppressed myotube formation, which was cancelled by PSL‐mediated M1‐to‐M2 macrophage polarization, as observed in the M1>M2‐CM group, which had similar myotube formation to that of the DM group.

### 2.3 Preparation and Characterization of PSL‐Multilayer‐Coated Samples

**Figure 3:**
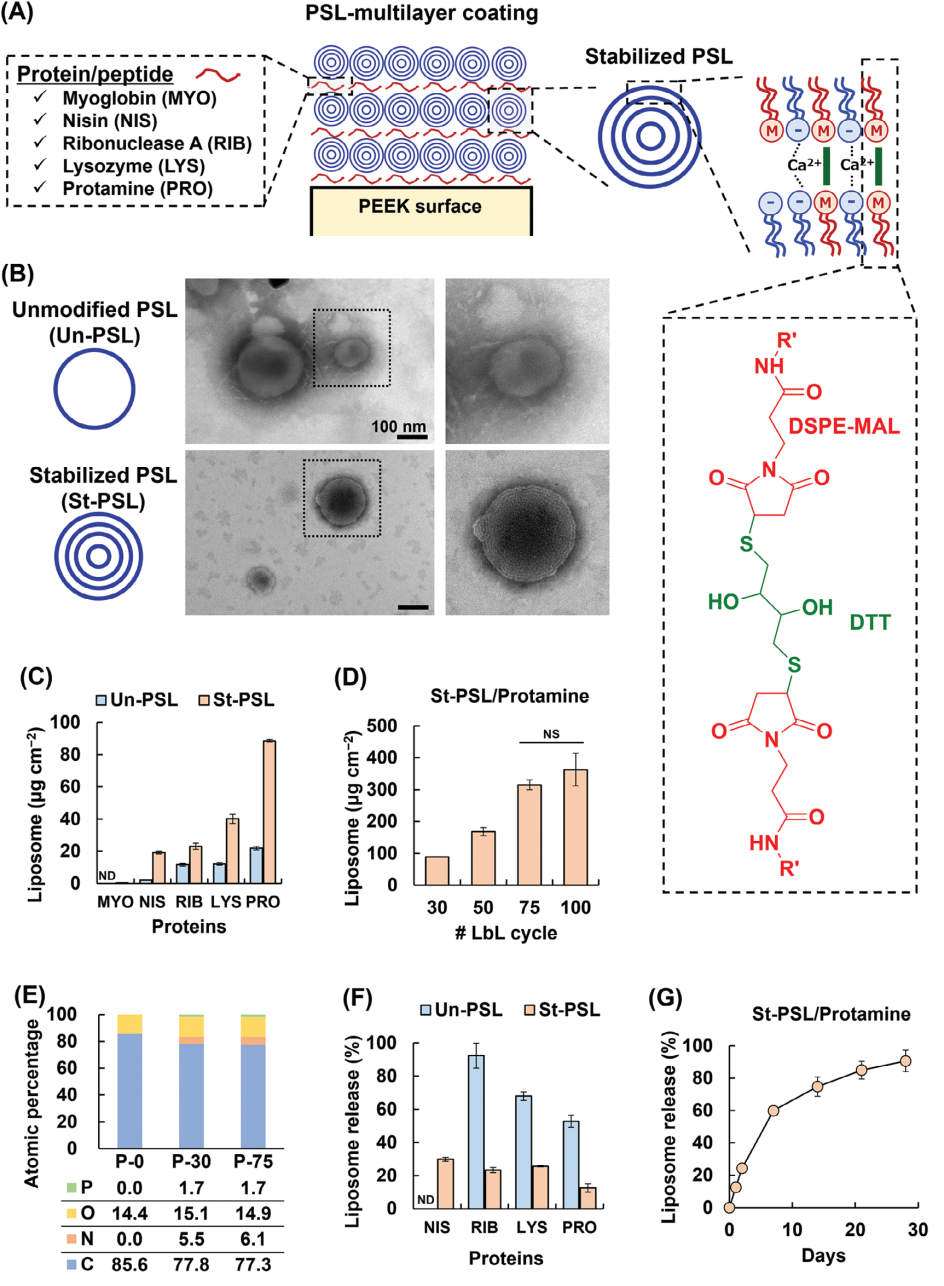
Characterization of phosphatidylserine‐containing liposomes (PSLs)‐coated poly(ether‐ether‐ketone) (PEEK). A) Schematic illustration of the preparation of PSLs‐coated PEEK using layer‐by‐layer (LbL) assembly based on electrostatic interactions between negatively charged PSLs and polycations. B) TEM images of unmodified unilamellar PSL (Un‐PSL) and multilamellar, crosslinked interbilayer PSL (St‐PSL). C) Effect of polycation on PSL multilayering. Number of LbL cycle is 30. Isoelectric points of polycations used are summarized in Table 1. MYO, Myoglobin; NIS, Nisin; Ribonuclease A, RIB; Lysozyme, LYS; Protamine, PRO; ND, not determined. D) Amount of PSLs modified on sample. NS, not statistically significant. E) Atomic composition of surfaces determined by X‐ray photoelectron spectroscopy. P‐x, where x is number of LbL cycles. F) PSL release from samples at 24 h after immersing in Hank's balanced salt solution (HBSS). G) Liposome release profile in HBSS. In D) and G), St‐PSL and protamine were used for PSL multilayering. Data are presented as mean ± SD (*n* = 3 or 4). Two‐tailed Student's *t*‐test was used in D).

Motivated by the promising in vitro results for preventing FBRs and promoting tissue regeneration by PSLs, PSL‐multilayers were constructed on PEEK surfaces using layer‐by‐layer (LbL) assembly based on electrostatic interactions between negatively charged PSLs (zeta‐potential: −49.3 mV) and natural polycations (i.e., protein and peptide) (**Figures** **3**A and S6, Supporting Information). Two types of PSL were tested (Scheme; Figure S7, Supporting Information): an original unilamellar PSL (unmodified PSL; Un‐PSL) and a multilamellar PSL with a crosslinked structure (stabilized PSL; St‐PSL). These lamellar structures were confirmed by transmission electron microscopy (TEM) images (Figure 3B). St‐PSL showed no cytotoxicity toward RAW 264.7 and C2C12 cells (Figure S8, Supporting Information). In addition, five types of polycations with different isoelectric points (pI) were tested as counterions for PSLs (**Table** **1**). Samples were prepared with 30 LbL cycles, and liposomes (labeled with fluorescein lipids) were quantified using the fluorescence intensity (Figure 3C). Regardless of the polycations tested, more liposomes were detected in St‐PSLs than in Un‐PSLs. This is because Un‐PSL disrupts the vesicle structure upon adsorption on a positively charged surface.^[^ ^39^, ^40^ ^]^ The amount of PSLs increased as the pI of the polycations increased. The best PSL modification was observed with protamine, which has the highest pI among the polycations tested. However, a PSL multilayer did not form with myoglobin, which has a nearly neutral charge. This suggests that polycations should have a high density of net positive charges for efficient PSL multilayering.

**Table 1:** Proteins or peptide used for preparation of liposome‐coated samples.

Name	Symbol	Isoelectric point
Myoglobin	MYO	7.3^a)^
Nisin	NIS	8.8^[^ ^73^ ^]^
Ribonuclease A	RIB	9.6^[^ ^74^ ^]^
Lysozyme	LYS	10.7^[^ ^75^ ^]^
Protamine	PRO	12−13^[^ ^76^ ^]^

^a)^ Data from the suppliers.

The multilayers prepared by combining St‐PSLs with protamine were further characterized. The amount of PSLs increased as the number of LbL cycles increased and reached a plateau at 75 LbL cycles (Figure 3D). Successful multilayering on PEEK was also confirmed by X‐ray photoelectron spectroscopy (XPS) (Figure 3E; Figure S9−S11, Supporting Information) and attenuated total reflection–Fourier transform infrared spectroscopy (ATR–FTIR) analyses (Figure S12, Supporting Information). In particular, the spectra contained new peaks ascribed to phospholipids and protamine in PSL‐modified PEEK. The original PEEK surface was hydrophobic (water contact angel of 84°) but became hydrophilic (43°) after it was coated with PSL‐multilayers owing to the charges of PSLs and protamine (Figure S13, Supporting Information). The coating did not change the original surface roughness (Figure S14, Supporting Information).

Liposome release was measured 24 h after the samples were immersed in Hank's balanced salt solution (Figure 3F). Depending on the polycations used, 50−90% of PSLs were released from the multilayers of Un‐PSLs. However, such burst release was suppressed (10−30% release) from the multilayers of St‐PSLs, with less PSL release as the pI of the paired polycation increased. Electrostatic interactions between PSLs and polycations are stronger as the pI of the polycation increases, hence slower release. The multilayer consisting of St‐PSLs and protamine provided sustained PSL release over a long timescale, namely one month (Figure 3G).

**Figure 4:**
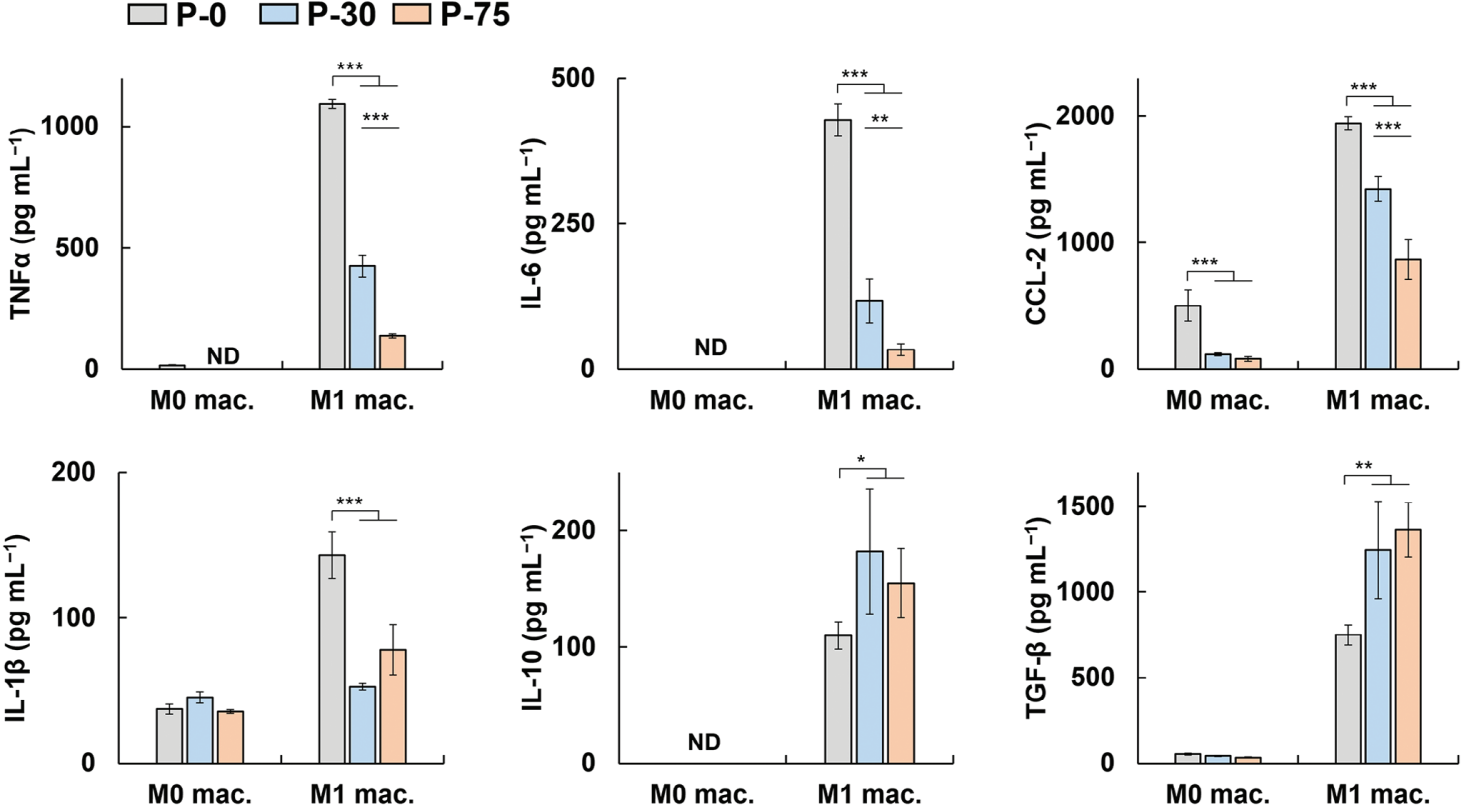
Reprogramming of M1 macrophages to M2‐like macrophages on phosphatidylserine‐containing liposome (PSL)‐multilayer‐coated samples. After culturing M0 or M1 RAW 264.7 macrophages on the samples for 24 h, cytokine concentrations were determined. P‐0, original PEEK; P‐30 and P‐75, PEEK coated using layer‐by‐layer assembly for 30 and 75 cycles, respectively. Data are presented as mean ± SD (*n* = 5). One‐way ANOVA with Tukey's multiple comparison tests were used. *p < 0.05; **p < 0.01; ***p < 0.001; ND, not determined (below the detection limit).

On the basis of these results, samples prepared with 30 and 75 LbL cycles of St‐PSL and protamine (hereafter denoted P‐30 and P‐75, respectively) were used for cell and animal experiments. The secretions of M0‐ and M1‐type macrophages cultured on the samples were analyzed (**Figure** **4**; Figure S15, Supporting Information). The bare substrate (P‐0) induced M0 macrophages to produce TNFα and CCL‐2, indicating that the PEEK surface induced a weak inflammatory response in M0 macrophages, consistent with previous reports.^[^ ^33^, ^35^ ^]^ However, the levels of these cytokines were diminished in PSL‐modified P‐30 and P‐75. In addition, in agreement with Figure 1A, P‐30 and P‐75 did not activate M0 macrophages to produce the M2‐type cytokines (IL‐10 and TGF‐β). As expected, M1 macrophages cultured on P‐0 produced cytokines and nitric oxide (NO) in higher levels than M0 counterparts. However, when cultured on P‐30 and P‐75, fewer inflammatory mediators (IL‐1β, IL‐6, TNFα, CCL‐2, and NO) and more anti‐inflammatory cytokines (IL‐10 and TGF‐β) were produced, which is the typical secretion profile of M2 phenotypes. The levels of TNFα, IL‐6, and CCL‐2 were significantly lower for P‐75 than for P‐30, which was likely associated with more PSLs on P‐75 than for P‐30 (Figure 3D). Surface hydrophilicity is a considerable factor that modulates cytokine secretions and even macrophage phenotypes.^[^ ^3^ ^]^ However, in our previous report, PEEK with different hydrophilicity showed subtle changes in TNFα and IL‐10 secretion from M1 macrophages compared to unmodified PEEK.^[^ ^35^ ^]^ These results indicate that while PSL‐multilayer coatings significantly alter the surface hydrophilicity (Figure S13, Supporting Information), the observed changes in cytokine profiles are mediated mainly by the PS eat‐me signaling.

### 2.4 PSL‐Multilayer Prevents FBRs while Facilitating Muscle Regeneration

**Figure 5:**
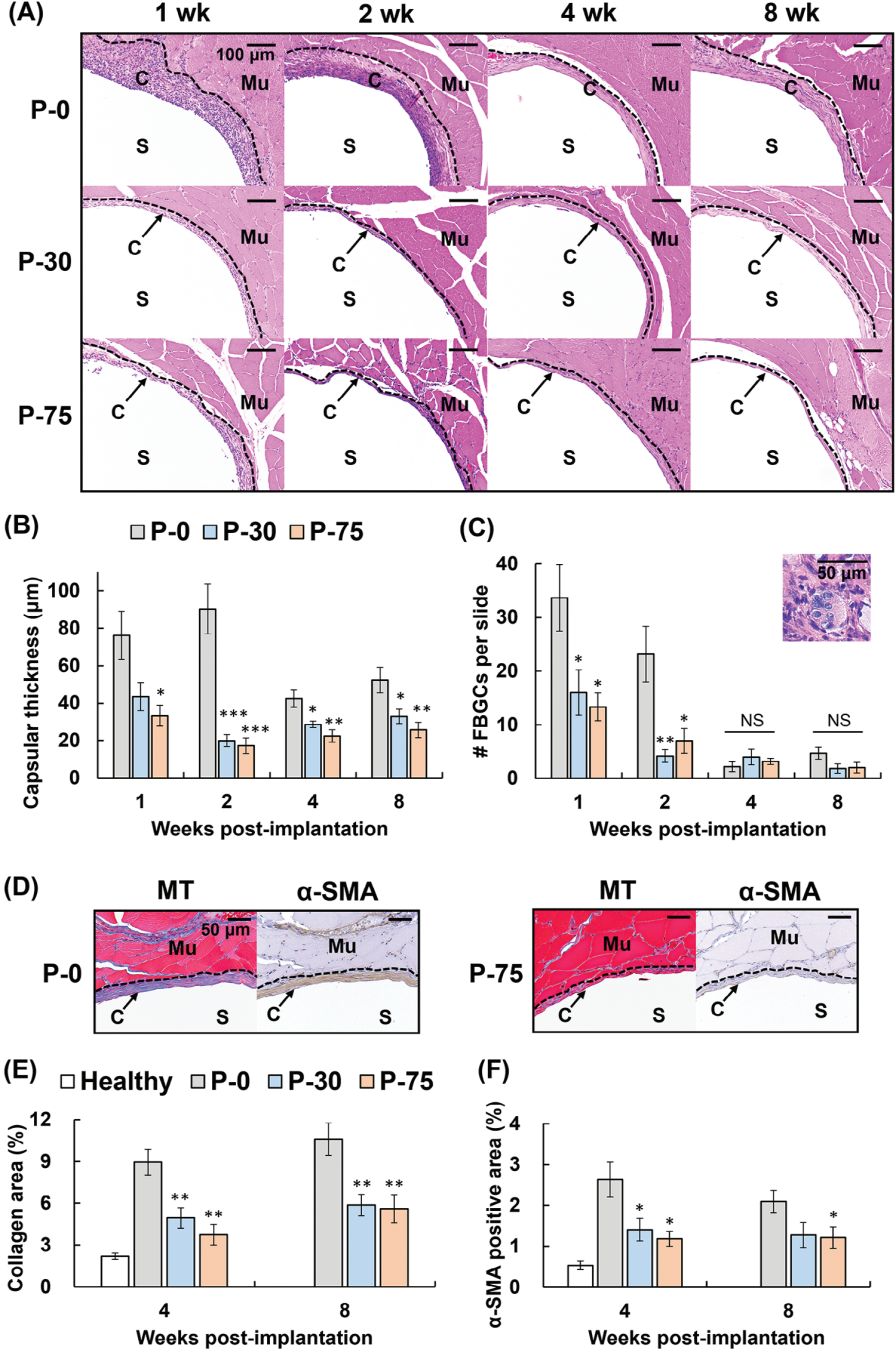
Histological observation of foreign body reactions to liposome multilayer‐coated poly(ether‐ether‐ketone) (PEEK) rods. A) Representative images of hematoxylin & eosin (HE)‐stained sections at 1−8 weeks post‐implantation. Scale bars = 100 µm. P‐0, original PEEK; P‐30 and P‐75, PEEK coated using layer‐by‐layer assembly for 30 and 75 cycles, respectively. S, sample; Mu, muscle; C, capsular formation; dashed line, interface between muscle and capsular formation. B) Capsular thickness. C) Number of foreign body giant cells (FBGCs) per slide and their representative image. Scale bars = 50 µm. D) Representative images of Masson's trichrome (MT)‐stained sections and α‐smooth muscle actin (α‐SMA; a myofibroblast marker)‐stained sections at 4 weeks post‐implantation. Collagen‐rich fibrotic tissue is stained in blue. Scale bars = 50 µm. E) Fibrotic area. F) α‐SMA‐positive area. Data are presented as mean ± SEM (*n* = 6). One‐way ANOVA with Tukey's multiple comparison tests were used. *p < 0.05; **p < 0.01; ***p < 0.001 (vs P‐0).

PEEK rods were inserted into rat skeletal muscles, and tissue responses were analyzed histologically at 1−8 weeks. Original PEEK (P‐0) induced typical FBRs characterized by thick capsular formation, abnormal inflammatory cell recruitment, and FBGC generation (**Figure** **5**A–C). Moreover, massive collagen deposits (stained in blue) were detected surrounding P‐0 at the later stage (4 and 8 weeks) (Figure 5D,E). Additionally, α‐smooth muscle actin (α‐SMA) immunohistochemistry revealed the high level of α‐SMA^+^ myofibroblasts in the fibrotic tissues, which are responsible for the synthesis of abnormal extracellular matrix (Figure 5D,F). However, both P‐30 and P‐75 strikingly prevented these series of FBRs with identical efficacy.

**Figure 6:**
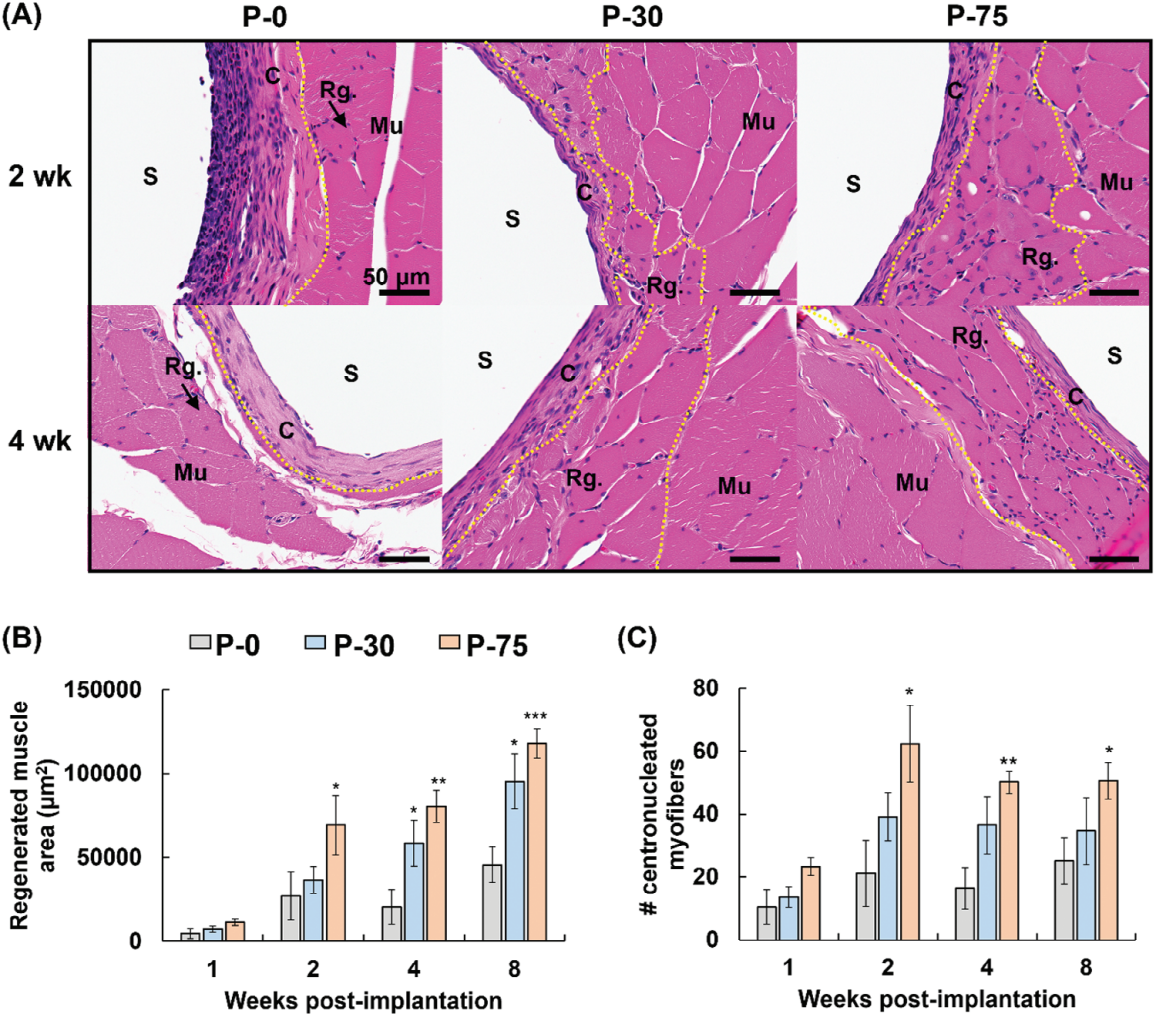
Histological observation of muscle healing surrounding liposome multilayer‐coated poly(ether‐ether‐ketone) (PEEK) rods. A) Representative images of hematoxylin & eosin (HE)‐stained sections (original magnification 400×). Scale bars = 50 µm. Centrally nucleated myofibers are considered regenerated and areas marked “Rg.” are rich in regenerated myofibers. S, sample; dashed yellow line, interface between regenerated muscle (Rg.) and capsular formation (C) or original muscle (Mu). P‐0, original PEEK; P‐30 and P‐75, PEEK coated using layer‐by‐layer assembly for 30 and 75 cycles, respectively. B) Sum of regenerated myofiber areas. C) Number of regenerated myofibers. Data are presented as mean ± SEM (*n* = 6). One‐way ANOVA with Tukey's multiple comparison tests were used. *p < 0.05; **p < 0.01; ***p < 0.001 (vs P‐0).

De novo muscle regeneration was investigated by examining regenerated myofibers with centrally positioned nuclei (**Figure** **6**A). In one week, the area (Figure 6B) and number (Figure 6C) of regenerated myofibers were similar among the samples. In two, four, and eight weeks, PSL‐modified samples, particularly P‐75, enhanced the regeneration of myofibers compared with bare samples (P‐0). Upon muscle injury, skeletal muscle satellite cells (smSCs) travel to the damaged tissue and eventually fuse with each other to form new myofibers. A number of reports suggest anti‐inflammatory drugs delay healing in part by inhibiting smSC migration.^[^ ^41^, ^42^ ^]^ However, PSLs did not change Pax‐7^+^ smSC recruitment surrounding the implantation site in one and two weeks (Figure S16, Supporting Information).

### 2.5 PSL‐Multilayer Promotes M1‐to‐M2 Macrophage Polarization In Vivo

**Figure 7:**
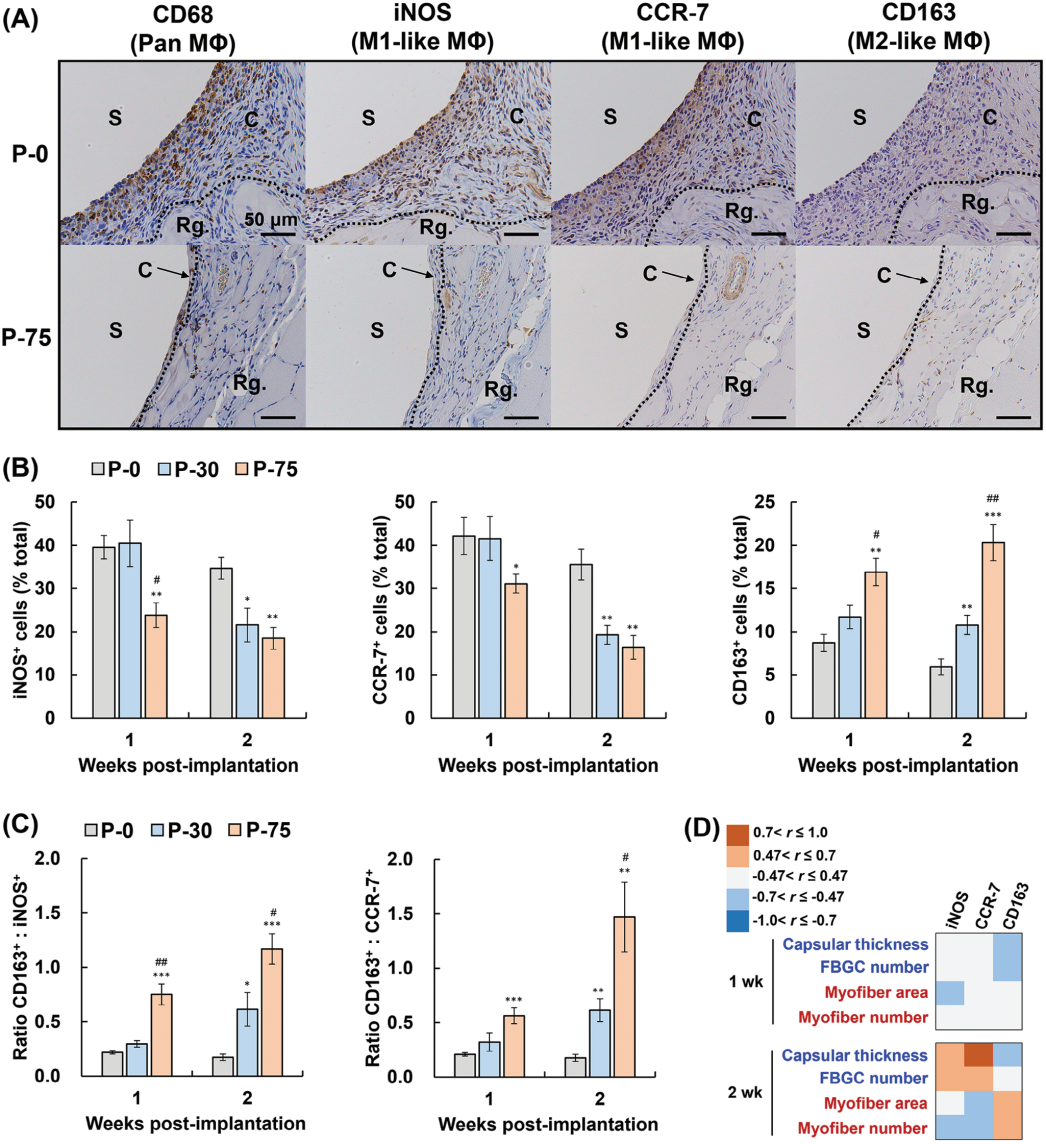
Macrophage phenotypes surrounding liposome multilayer‐coated poly(ether‐ether‐ketone) (PEEK) rods. A) Representative images of inducible nitric oxide synthase (iNOS), C‐C chemokine receptor type 7 (CCR‐7), and CD163‐stained histological sections at 2 weeks post‐implantation (original magnification 400×). Scale bars = 50 µm. iNOS and CCR‐7 are markers of M1 macrophages, and CD163 is a marker of M2 macrophages. P‐0, original PEEK; P‐30 and P‐75, PEEK coated using layer‐by‐layer assembly for 30 and 75 cycles, respectively. B) Percentage of M1‐like (iNOS^+^ or CCR‐7^+^ cells) and M2‐like macrophages (CD163^+^ cells) over total cells (*n* = 6). C) Ratio M2:M1 macrophages (*n* = 6). Data are presented as mean ± SEM. One‐way ANOVA with Tukey's multiple comparison tests were used. *p < 0.05; **p < 0.01; ***p < 0.001 (vs P‐0), ^#^ *p* < 0.05; ^##^ *p* < 0.01 (vs P‐30). Representative images of other samples can be found in Figures S18 and S19, Supporting Information. D) Pearson correlation coefficient (*r*) between macrophages and tissue responses. Raw data are in Table S1, Supporting Information. Owing to the number of samples, samples with *r* values between –0.47 and +0.47 have *p* values above 0.05.

Macrophage infiltration and their phenotypes were determined by immunohistochemistry using CD68 (pan macrophages), inducible nitric oxide synthase (iNOS; M1 macrophages), CCR‐7 (M1 macrophages), and CD163 (M2 macrophages). In all samples, the levels of CD68^+^ cells were maximal in one week and gradually decreased with time (Figure S17, Supporting Information). Compared with P‐0, P‐30 and P‐75 impaired the infiltration of CD68^+^ cells in one and two weeks. All samples had similar levels in four and eight weeks. Macrophage phenotypes were examined only in one and two weeks because of the small number of macrophages in four and eight weeks. iNOS^+^ or CCR‐7^+^ M1‐like macrophages were present mainly at the surface of the samples (**Figure** **7**A; Figures S18 and S19, Supporting Information). CD163^+^ M2‐like macrophages, which are essential for muscle repair, were distributed near regenerated myofibers, consistent with previous reports.^[^ ^43^, ^44^ ^]^ In the P‐0 group, iNOS^+^ or CCR‐7^+^ M1‐like macrophages were predominant over CD163^+^ M2‐like macrophages (Figure 7B,C). In the P‐75 group, however, PSLs induced M1‐to‐M2 macrophage polarization, and thus the levels of M1‐like macrophages decreased and the levels of M2‐like macrophages increased, resulting in the dramatic increase in the M2/M1 macrophage ratio. P‐30 also improved macrophage phenotypes, although not to the same level as P‐75 because the amount of PSLs in P‐30 was threefold lower than that in P‐75 (Figure 3D).

To determine the effects of macrophage phenotypes on tissue responses, Pearson correlation coefficients were calculated using data of macrophages and FBRs (i.e., capsular thickness and FBGC count) or muscle regeneration (i.e., total area and number of regenerated myofibers) (Figure 7D; Table S1, Supporting Information). Both capsular thickness and FBGC count in one week were negatively correlated with CD163^+^ M2‐like macrophages but had no correlation with iNOS^+^ or CCR‐7^+^ M1‐like macrophages. In two weeks, CD163^+^ cells followed the same trend as the capsular thickness in one week. iNOS^+^ or CCR‐7^+^ cells had a strong positive correlation with capsular thickness and FBGC count. Meanwhile, muscle regeneration in one week was comparable among the samples as mentioned earlier (Figure 6), and hence, correlations were indiscernible. In two weeks, however, a clear relationship was detected: Muscle regeneration was negatively correlated with iNOS^+^ or CCR‐7^+^ M1‐like macrophages, but CD163^+^ M2‐like macrophages had the opposite trend to M1‐like macrophages. Taken together, the in vivo study demonstrated that M1‐like macrophages boosted FBRs against implanted materials and impeded muscle regeneration, while M2‐like macrophages vice versa.

## 3 Discussion

Clinically used anti‐inflammatory drugs, which have been extensively studied to prevent FBRs, have no ability to target cells and thus adversely affect not only host tissues but also tissue repair following injury.^[^ ^16^, ^17^, ^18^, ^19^, ^20^, ^21^, ^22^, ^23^, ^24^ ^]^ For instance, in skeletal muscles, dexamethasone causes atrophy of C2C12 myotubes and muscle tissue by triggering the ubiquitin–proteasome system to degrade proteins associated with muscle mass.^[^ ^20^ ^]^ NSAIDs inhibit mainly cyclooxygenases 1 and 2 (COX1/2) and delay muscle repair by compromising smSC and myoblast responses.^[^ ^16^, ^21^, ^22^, ^23^, ^24^ ^]^ Here, PSLs were found to have no observable detrimental effects on C2C12 myoblasts, smSCs, and muscle tissues most likely because of their macrophage targeting ability. Furthermore, other approaches, including modifying surface chemistry or physical property of implantable devices, have also been employed to minimize the FBR. Non‐specific protein adsorption and inflammatory cell infiltration and adhesion stimulate the process of FBR.^[^ ^45^ ^]^ Neutral polymers^[^ ^46^ ^]^ and zwitterionic polymers^[^ ^47^ ^]^ create anti‐fouling bioinert surfaces on the wide range of materials. Despite the encouraging reports for FBR mitigation, blocking adhesion of host cells, particularly stem/progenitor cells, results in poor outcomes in tissue repair and biomaterial‐tissue integration. Although implants with the optimal surface topography^[^ ^48^ ^]^ and stiffness^[^ ^49^ ^]^ induce the FBR mitigation through modulation of immune cell and fibroblast responses, surface physical/chemical parameters also have a significant impact on not only immune infiltrates but also stem/progenitor/parenchymal cells.^[^ ^50^ ^]^ Therefore, it may be theoretically difficult to find the optimal surface that not only prevents FBR but also promotes tissue repair. However, our study demonstrates that PSL is a useful means to prevent the implant‐induced fibrosis and to promote the tissue regeneration.

Given the causal role of activated macrophages in FBRs and many diseases involving inflammation and fibrosis,^[^ ^51^, ^52^, ^53^, ^54^ ^]^ it is reasonable to assume that the abnormal polarization of resident naïve macrophages, which exist in major tissues throughout the body, is a potential risk factor. Although M2‐inducible cytokines (e.g., IL‐4) delivered from biomaterials have been shown to improve tissue repair,^[^ ^55^, ^56^ ^]^ these cytokines strongly stimulate naïve M0 macrophages to increase the production of cytokines and specific surface molecules in response to a given stimuli, leading to potential adverse effects (as discussed later).^[^ ^7^, ^38^, ^39^ ^]^ In this regard, PSLs offer a significant advantage that they specifically react with M1 macrophages, not M0 macrophages, to polarize M1 into M2‐like phenotypes. This suggests that PSL is a safer therapeutic agent than known cytokines triggering M2 macrophages.

A well‐organized macrophage response that starts with an M1‐like phenotype in an early phase followed by a shift into an M2‐like phenotype during the repair phase is considered essential for the repair of various tissues.^[^ ^41^, ^42^ ^]^ Nevertheless, NSAIDs‐mediated COX1/2 inhibition diminishes or delays M1‐to‐M2 macrophage polarization,^[^ ^16^, ^57^, ^58^ ^]^ which also accounts for compromised tissue repair seen in NSAIDs‐treated rodents and humans. Here, samples coated with PSL‐multilayers were biased toward M2‐like phenotypes during macrophage polarization, which coincided with improved tissue repair. M1 macrophages produce inflammatory cytokines that are beneficial for myoblast proliferation, although they also induce muscle damages by secreting cytotoxic levels of reactive oxygen/nitrogen species (e.g., NO).^[^ ^43^, ^59^, ^60^ ^]^ Chronic inflammation driven by M1 macrophages is also implicated in muscle wasting and loss of myogenic differentiation. In contrast, effector cytokines secreted by M2 macrophages deactivate M1 macrophages and direct smSCs and myoblasts toward muscle regeneration.^[^ ^43^, ^60^ ^]^ These reports support the following finding of this study: Differentially activated macrophages induced diverse C2C12 responses, with secretions from M1 macrophages promoting proliferation yet preventing migration and myogenic differentiation, while secretions from PSL‐induced M2‐like macrophages induced opposite trends.

Fusion of macrophages into FBGCs and their role in dissemination of fibrous capsule formation are hallmark of FBRs.^[^ ^3^, ^4^, ^5^, ^6^ ^]^ Nevertheless, there are limited agents that can inhibit the multinucleation of macrophages. However, PSLs were found to suppress the generation of multinucleated giant cells and FBGCs. IL‐4 and/or IL‐13 are considered the key driver of FBGC generation. This study demonstrated IL‐4 increased the macrophage production of CCL‐2, which promotes the formation of FBGCs and multinucleated giant cells,^[^ ^37^, ^38^ ^]^ but PSLs significantly decreased the IL‐4‐induced CCL‐2 expression and consequently inhibited multinucleated giant cell formation.

Imbalanced populations of M1 and M2 macrophages are associated with the biomaterials‐induced fibrosis and fibrotic diseases.^[^ ^3^, ^4^, ^5^, ^6^, ^7^, ^51^, ^52^, ^53^, ^54^ ^]^ FBRs and consequent fibrotic capsule formation are normally overwhelmed by acute and chronic M1‐biased macrophages, which aggressively accumulate on the surface of engrafted biomaterial.^[^ ^61^, ^62^, ^63^, ^64^ ^]^ Here, bare samples consistently triggered thick fibrotic scar formation with the massive accumulation of iNOS^+^ or CCR‐7^+^ M1‐like macrophages together with α‐SMA^+^ myofibroblasts on the sample surface. However, the PSL‐multilayer coating succeeded in minimizing multiple FBR events by improving the M1/M2 balance and reducing macrophage infiltration. The anti‐fibrotic effect of PSL‐based therapeutics has also been observed in a mouse model of cardiac fibrosis, suggesting an anti‐fibrotic role of PSL‐induced M2 macrophages.^[^ ^29^ ^]^ This study also demonstrated that the FBR was negatively correlated with the degree of tissue repair. Suppression of FBRs at early time points is important in scaffolds for tissue regeneration or in medical devices (e.g., stents and neural interfaces) that require tissue repair to achieve maximum function or to avoid side effects (e.g., in‐stent restenosis).^[^ ^65^, ^66^, ^67^, ^68^ ^]^ The inflammatory environment dominated by the inflammatory infiltrates, including macrophages and FBGCs, damages cells/tissues and inhibits tissue repair by suppressing cell differentiation as described earlier. Functional tissue repair is also inhibited by the fibrous tissue, which is formed at a place where parenchymal tissue was originally located.

M1 macrophages produce cytokines (e.g., TNFα, IL‐1β, and IL‐6) that are involved in the development and progression of fibrosis.^[^ ^4^, ^5^, ^6^, ^7^, ^51^, ^52^, ^53^, ^54^ ^]^ M2‐inducing cytokines (IL‐4 and IL‐13) and M2‐producing growth factors (e.g., TGF‐β, platelet‐derived factor‐BB) are also characteristics of fibrosis, although specific M2 macrophage populations function as anti‐fibrotic phenotypes by de‐differentiating or inactivating scar‐forming myofibroblasts via IL‐10 or activation of matrix metalloproteinases (fibrolytic enzymes).^[^ ^7^, ^51^, ^52^, ^53^, ^54^, ^69^, ^70^ ^]^ M2 macrophages are generally subdivided into IL‐4‐ and/or IL‐13‐induced M2a, IL‐10‐stimulated M2c, and others.^[^ ^7^ ^]^ The result using a 3D co‐culture model of human cells suggests that M2a drives myofibroblasts to deposit dense, thick, and highly elastic collagen, while M2c shows an inhibitory effect and rather functions as an anti‐fibrotic subtype.^[^ ^69^ ^]^ Additionally, the transfer of M2c into nephrotic mice dramatically reduces renal fibrosis.^[^ ^71^ ^]^ Furthermore, CD163 is used as a marker for M2c,^[^ ^72^ ^]^ and this study confirmed that the early increase in CD163^+^ macrophages induced by PSLs suppressed the development of fibrosis associated with myofibroblast recruitment. These lines of evidence suggest that PSL‐induced M2 macrophages may have anti‐fibrotic activity and share this function with M2c macrophages, but further comprehensive studies are needed to test this hypothesis.

There are potential limitations to the present study. First, the magnitude of FBRs and tissue repair depends on biomaterial characteristics such as size, shape, surface chemistry, stiffness, and topography, and further study is needed for the application of our multilayer coating to various biomaterials.^[^ ^3^, ^4^, ^5^, ^6^, ^7^ ^]^ However, it is reasonable to assume that when our coating technology is applied to other devices or materials, similar effects may be obtained because our strategy, which induces M1‐to‐M2 macrophage polarization, is effective to reduce FBRs and to stimulate tissue healing. In fact, M1 promotes fibrotic encapsulation during FBRs and incomplete tissue repair in various tissues, and vice versa for M2 as discussed earlier. Second, as mentioned above, the present results may suggest that PSL‐induced M2 macrophages are somewhat similar to the M2c subtype from the perspective of their anti‐fibrotic activities. However, this study and previous investigations have not characterized M2 subtypes in vivo nor fully addressed the relevance of M2 subtypes to tissue responses because no specific markers are currently available to determine M2 subtypes in vivo. To this end, advanced technologies, such as mass‐cytometry and single‐cell RNA sequencing, would be useful to elucidate the plasticity and dynamics of the macrophage population as well as other cells participating in different stages of tissue repair.^[^ ^6^ ^]^ These understandings are of interest for future research and may advance the PSL‐based therapeutic strategy as well as the development of new immune‐targeted biomaterials.

## 4 Conclusion

The PSL‐multilayer coating was constructed on PEEK surfaces using the layer‐by‐layer assembly of chemically stabilized PSLs and oppositely charged proteins, allowing the long‐term release of PSLs. In rat muscles, bare samples produced a chronic inflammatory microenvironment characterized by a high density of CCR‐7^+^ or iNOS^+^ M1‐like macrophages and α‐SMA^+^ myofibroblasts coupled with sparse CD163^+^ M2‐like macrophages, resulting in intense FBRs and scarring with limited tissue repair. The PSL‐multilayer coating, however, resisted FBRs and fibrosis while accelerating muscle regeneration by improving the M1/M2 balance. To our knowledge, our study is the first to demonstrate the effectiveness of PSL‐based surface modification of implantable medical devices for the production of an anti‐FBR, anti‐fibrotic, pro‐healing coating.

## 5 Experimental Section

### Preparation and Characterization of Liposomes‐Coated PEEK Substrate

PSLs and multilamellar PSLs with cross‐linked adjacent phospholipids were prepared as previously reported.^[^ ^31^ ^]^ The detailed procedures are described in Supporting Information. PEEK substrates (10‐mm square with 1‐mm thickness for characterizations and cell experiments or 1‐mm Φ cylinder with 10‐mm length for animal experiments) were washed ultrasonically with acetone, ethanol, and ultrapure water and then dried in vacuo. The surfaces were treated with air plasma using a plasma surface treatment processor (CUTE‐MP, Femto Science, Gyeonggi‐Do, Korea) operated at 50 kHz and 100 W with an airflow of 10 sccm under 0.05 Torr for 3 min. The resulting substrates were quickly immersed in 1 mg mL⁻^1^ solution of protein or peptide (listed in Table 1). Liposomes were coated onto PEEK substrates using the LbL method.^[^ ^31^ ^]^ As counterions of PSL (possessing negative charges) in the LbL process, polycations,^[^ ^73^, ^74^, ^75^, ^76^ ^]^ including myoglobin (from equine; Serva Electrophoresis, Heidelberg, Germant), nisin (from *Lactococcus lactis*; Sigma‐Aldrich, St. Louis, MO, USA), ribonuclease A (from bovine pancreas; Fujifilm Wako Pure Chemical, Osaka, Japan), lysozyme (from egg white, Fujifilm Wako Pure Chemical), and protamine sulfate (from salmon; Sigma‐Aldrich), were used. A single LbL cycle consisted of substrate immersion in the above polycation solution (1 mg mL^−1^, 1 min), substrate washing with ultrapure water, substrate immersion in a liposome solution (1 mg mL^−1^, 1 min), and substrate washing with ultrapure water. After the final LbL step, the substrates were dried in vacuo in the dark. Samples were characterized as previously reported,^[^ ^31^ ^]^ with details in Supporting Information.

### Cells

RAW 264.7 macrophages (RIKEN BioResource Research Center, Ibaraki, Japan) were maintained in Eagle's minimum essential medium (EMEM; Fujifilm Wako Pure Chemical) supplemented with 10% v/v fetal bovine serum (FBS; Thermo Fisher Scientific), 1% v/v non‐essential amino acids solution (Fujifilm Wako Pure Chemical) and 1% v/v penicillin‐streptomycin (P/S; Fujifilm Wako Pure Chemical). C2C12 murine skeletal muscle myoblasts (RIKEN) were maintained in Dulbecco's modified Eagle's medium (DMEM; Fujifilm Wako Pure Chemical) supplemented with 10% v/v FBS and 1% v/v P/S. NIH/3T3 murine fibroblasts (RIKEN) were maintained in DMEM supplemented with 10% v/v bovine serum (Thermo Fisher Scientific) and 1% v/v P/S.

### Uptake, Cytotoxicity, and Anti‐Inflammatory Studies

Cellular uptake of PSL was determined using a fluorescence‐based method as previously reported.^[^ ^27^ ^]^ The cytotoxicity of PSL was determined using Cell‐Counting Kit‐8 (CCK‐8; Dojindo, Kumamoto, Japan), and the anti‐inflammatory assay (cytokines and nitric oxide [NO]) was conducted as previously reported.^[^ ^30^, ^31^ ^]^ Briefly, M0 macrophages were stimulated with 30−100 µg mL^−1^ PSL or 25 ng mL^−1^ IL‐4 (Miltenyi Biotec, Gladbach, Germany). For M1 macrophages, cells were exposed to a mixture of 100 ng mL^−1^ lipopolysaccharide (LPS; Sigma‐Aldrich) and PSL or IL‐4. For M2 macrophages, cells were cultured in 50 ng mL^−1^ IL‐4 and/or 30−100 µg mL^−1^ PSL. After 24 h of activation, the media were collected and frozen at −80 °C until measurement. Cytokine levels were measured using a series of uncoated ELISA Kit (Thermo Fisher Scientific), Mouse IL‐10 ELISA Kit, (Proteintech Group Inc., Rosemont, IL, USA) or Mouse TGF‐beta1 ELISA Kit (Proteintech Group Inc.) according to the manufacturer's instructions. To estimate the NO concentration, the stable metabolite of NO, nitrite, was quantified using the Griess–Romijn nitrite reagent (Fujifilm Wako Pure Chemical). The absorbance was measured using iMark microplate reader (Bio‐Rad Laboratories, Hercules, CA, USA).

### Multinucleated Giant Cell Formation

RAW 264.7 macrophages were seeded in 24‐well plates at an initial density of 5 000 cells/well. After 24 h, multinucleated giant cell formation was initiated by replacing the medium containing 25 ng mL^−1^ IL‐4 and/or 10−100 µg mL^−1^ PSL. The medium was changed every two days. On day six, multinucleated giant cells were counted. Cells were fixed by 4% paraformaldehyde/PBS (Fujifilm Wako Pure Chemical) for 10 min and then permeabilized in PBS containing 0.5% v/v Triton X‐100 for 5 min. F‐actin was stained with Acti‐stain 488 phalloidin (Cytoskeleton, Inc., Denver, CO, USA) for 30 min in the dark. Nuclei were counterstained with Hoechst 33 342 (Dojindo, Kumamoto, Japan). Cells with three or more nuclei were defined as multinucleated giant cells. Images were acquired and analyzed using ZOE Fluorescence Cell Imager (Bio‐Rad Laboratories) and ImageJ (National Institutes of Health, Bethesda, MD, USA).

### Cellular Responses in Macrophage CM

CM was prepared as previously reported.^[^ ^31^ ^]^ Proliferation, migration, and/or differentiation of C2C12 and NIH/3T3 cells were tested as previously reported with minor modification.^[^ ^16^ ^]^ For the collagen synthesis assay, NIH/3T3 fibroblasts were seeded in 24‐well plates at an initial density of 25 000 cells/well. After culturing for 24 h, the culture medium was replaced with DMEM supplemented with 10% v/v bovine serum, 1% v/v P/S, and/or 25% v/v macrophage CM. The medium was refreshed every two days. On day ten, collagen was stained using Sirius Red/Fast Green Collagen Staining Kit (Chondrex, Inc., Woodinville, WA, USA) according to the manufacturer's instructions. Images were acquired and analyzed using All‐in‐One Fluorescence Microscopy (BZX710; Keyence, Osaka, Japan) and ImageJ. The details are described in Supporting Information.

### Animal Experiments

All animal studies were approved by the ethics committee of the National Institute of Advanced Industrial Science and Technology (#A2017−A2019‐303) and performed in accordance with the Guidelines for Animal Experiments established by the Ministry of Health, Labour and Welfare of Japan. Specific pathogen‐free Sprague Dawley rats (male, 12‐weeks old) were purchased from Japan SLC (Shizuoka, Japan). The rats were single‐housed in standard cages and maintained in a temperature‐controlled room (22 °C) with a 12‐h light‐dark cycle. The rats were fed a regular diet (MF; Oriental Yeast, Tokyo, Japan) and provided tap water ad libitum. The rats were anesthetized with a mixture of medetomidine (0.75 mg kg^−1^), midazolam (4 mg kg^−1^), and butorphanol (5 mg kg^−1^). The fur surrounding the insertion site was shaved and cleaned with iodine before surgery. An incision (≈1.5 cm) was made along the axis of the femur to open the quadricep muscle. Samples (Φ1 mm × 10 mm) were inserted into quadricep muscles using a catheter (16G, Surflo F&F, Terumo, Tokyo, Japan). The skin was closed by suturing using a nylon suture, and the incision sites were cleaned with iodine. The rats were euthanized on days 7, 14, 28, and 56, and muscle with implants were harvested for routine histological analysis (please see Supporting Information).^[^ ^30^, ^31^ ^]^

### Statistical Analysis

Statistical analysis of differences between means was performed using a two‐tailed Student's *t*‐test or one‐way analysis of variance, followed by Tukey's multiple comparison test, with *p*‐values calculated manually using Excel. To determine if a Pearson's correlation coefficient (*r*) is statistically significant, the corresponding *t*‐score and *p*‐value were calculated. *p* < 0.05 was considered statistically significant. Owing to the data volume, a sample with −0.47 < *r* ≤ 0.47 had *p* > 0.05, and thus they had no correlation.

## Conflict of Interest

The authors declare the following financial interests/personal relationships which may be considered as potential competing interests: Riki Toita received a research grant from Niterra Co., Ltd. Masahiro Kitamura and Shinjiro Kasahara are employees of Niterra Co., Ltd. The remaining authors declare no competing interests.

## Author Contributions

Riki Toita: Conceptualization, Data curation, Formal analysis, Funding acquisition, Investigation, Methodology, Project administration, Writing – original draft, Writing – review & editing. Masahiro Kitamura: Methodology. Akira Tsuchiya: Investigation. Jeong‐Hun Kang: Methodology, Writing – review & editing. Shinjiro Kasahara: Conceptualization, Methodology. All authors read and approved the final manuscript.

## Supporting information

Supporting Information

## Data Availability

The data that support the findings of this study are available from the corresponding author upon reasonable request.
