# Supplementary material for: Releasable, Immune‐Instructive, Bioinspired Multilayer Coating Resists Implant‐Induced Fibrosis while Accelerating Tissue Repair
Source: Adv Healthc Mater. 2023 Dec 19;13(5):2302611. doi: 10.1002/adhm.202302611 (PMC11468989; doi:10.1002/adhm.202302611)
Supplement: Supplementary file 1 — Supporting Information [file ADHM-13-2302611-s001.pdf]

ADVANCED HEALTHCARE MATERIALS

Supporting Information

for *Adv. Healthcare Mater.*, DOI 10.1002/adhm.202302611

Releasable, Immune-Instructive, Bioinspired Multilayer Coating Resists Implant-Induced Fibrosis while Accelerating Tissue Repair

Riki Toita, Masahiro Kitamura, Akira Tsuchiya, Jeong-Hun Kang and Shinjiro Kasahara*

Supporting Information

Releasable, Immune-Instructive, Bioinspired Multilayer Coating Resists Implant-Induced Fibrosis while Accelerating Tissue Repair

Riki Toita,[1,2] Masahiro Kitamura,[3,4] Akira Tsuchiya,[5] Jeong-Hun Kang,[6] and Shinjiro Kasahara[3]*

[1] Biomedical Research Institute, National Institute of Advanced Industrial Science and Technology (AIST), 1-8-31 Midorigaoka, Ikeda, Osaka, 563-8577, Japan
E-mail: toita-r@aist.go.jp

[2] AIST-Osaka University Advanced Photonics and Biosensing Open Innovation Laboratory, AIST, 2-1 Yamadaoka, Suita, Osaka 565-0871, Japan

[3] Niterra Co., Ltd., 2808 Iwasaki, Komaki, Aichi 485-8510, Japan

[4] NGK Spark Plug-AIST Healthcare Materials Cooperative Research Laboratory, 2266-98 Anagahora, Shimoshidami, Moriyama-ku, Nagoya, Aichi 463-8560, Japan

[5] Department of Biomaterials, Faculty of Dental Science, Kyushu University, 3-1-1 Maidashi, Higashi-ku, Fukuoka 812-8582, Japan

[6] Division of Biopharmaceutics and Pharmacokinetics, National Cerebral and Cardiovascular Center Research Institute, 6-1 Shinmachi, Kishibe, Suita, Osaka, 564-8565, Japan

Table of Contents

- ✓ **Supplemental Methods.**
- ✓ **Table S1. Pearson correlation coefficient.**
- ✓ **Figure S1. Anti-inflammatory effect of PSLs.**
- ✓ **Figure S2. Cellular uptake of PSLs.**
- ✓ **Figure S3. Cytotoxicity of PSLs.**
- ✓ **Figure S4. Myotube atrophy by PSLs and dexamethasone.**
- ✓ **Figure S5. Myogenic differentiation in the presence of PSLs.**
- ✓ **Figure S6. Scheme of the PSL-multilayer-coated PEEK.**
- ✓ **Figure S7. Scheme of a multilamellar, crosslinked interbilayer PSL.**
- ✓ **Figure S8. Cytotoxicity of St-PSLs.**
- ✓ **Figure S9. Wide-scan XPS spectra.**
- ✓ **Figure S10. High-resolution XPS spectra.**
- ✓ **Figure S11. Chemical structures.**
- ✓ **Figure S12. ATR-FTIR spectra.**
- ✓ **Figure S13. Water contact angle.**
- ✓ **Figure S14. Surface roughness.**
- ✓ **Figure S15. Nitric oxide production from macrophages.**
- ✓ **Figure S16. Recruitment of Pax-7$^+$ skeletal muscle satellite cells.**
- ✓ **Figure S17. Time course of CD68$^+$ macrophage recruitment.**
- ✓ **Figure S18. Macrophage phenotypes at 1 week.**
- ✓ **Figure S19. Macrophage phenotypes surrounding P-30 at 2 weeks.**
- ✓ **References**

Supplemental Methods

Preparation of Phosphatidylserine Liposomes (PSLs): Phospholipids were dissolved in chloroform:methanol (9:1) at the concentration of 10 mg mL^{-1}. PS (Sigma–Aldrich, St. Louis, MO, USA), phosphatidylcholine (PC; Sigma-Aldrich), and 1,2-distearoyl-*sn*-glycero-3-phosphoethanolamine-maleimide (DSPE-MAL; NOF, Tokyo, Japan) were mixed in the molar ratio of 3:2:5, and the organic solvent was removed under nitrogen to obtain lipid films. The lipid film was resuspended in Bis-Tris buffer (5×10^{-2} M, pH 7.1) to prepare 10 mg mL^{-1} liposome solutions. Liposomes were extruded through polycarbonate membranes with different pore sizes (800 nm and 200 nm) (Avanti Polar Lipids, Alabaster, AL, USA). The resulting liposomes were mixed with $CaCl_2$ solution (final concentration of 4×10^{-3} M) and dithiothreitol (DTT) solution (final concentration of 1 equivalent to maleimide groups), followed by incubation at room temperature for 30 min to prepare multilamellar liposomes with crosslinked adjacent phospholipids. The zeta-potential of liposomes was determined using Zetasizer NS (Malvern Instruments, Malvern, UK).

Characterization of the Liposomes-Coated Samples: The surface chemistry was analyzed using X-ray photoelectron spectroscopy (XPS; K-alpha, Thermo Fisher Scientific, East Grinstead, UK) and attenuated total reflection–Fourier transform infrared spectroscopy (ATR–FTIR; FT/IR-6200, JASCO, Tokyo, Japan). In the XPS analysis, the maximum peak of C 1s was set to 284.8 eV. The water contact angles (1.5 μL of water droplets) were measured using a contact angle meter (DM500, Kyowa

Interface Science, Saitama, Japan). The surface roughness was measured by laser scanning microscopy (Violet Laser VK-9700; Keyence, Osaka, Japan). Liposomes containing 3 mol% *N*-(fluorescein-5-thiocarbamoyl)-1,2-dihexadecanoyl-*sn*-glycero-3-phosphoethanolamine, triethylammonium salt (fluorescein DHPE) (Thermo Fisher Scientific, Waltham, MA, USA) were used to quantify liposomes modified on samples as previously reported.[S1] They were dissociated from the substrates and disrupted by shaking the samples in a buffer containing 1×10^{-1} M Tris-HCl (pH 7.5), 1 M sodium chloride, and 1% Triton-X100 for 2 days in the dark. To evaluate liposome release, liposome-modified samples were soaked in 1 mL of Hank's balanced salt solution and shaken at 500 rpm in the dark. The solution was collected at the prescribed time, and the fluorescence intensity was measured to quantify the released liposomes using a Synergy HT plate reader (BIO-TEK Instruments Inc., Winooski, VT, USA) with excitation (485/20 nm) and emission (530/25 nm) filters.

Anti-inflammatory Effects of PSLs on Human THP-1 Macrophages: THP-1 human monocytes (RIKEN BioResource Research Center, Ibaraki, Japan) were cultured in 24-well plates at an initial density of 50,000 cells/well and were allowed to differentiate into macrophages in RPMI-1640 containing 10% FBS, 1% antibiotics, and 1.5×10^{-7} M phorbol 12-myristate 13-acetate (Sigma-Aldrich) for 48 h. The macrophages were treated with a mixture of 100 ng mL^{-1} lipopolysaccharide (LPS; Sigma-Aldrich) and 30 or 100 μg mL^{-1} of PSLs. After 24 h, the medium was collected, and concentrations of TNFα and IL-6 were measured using ELISA kits (Thermo Fisher Scientific) following the manufacturer's instructions.

Myotube Atrophy: Myotube atrophy was examined as previously reported with minor modification.[S2] C2C12 cells were placed in a 24-well culture plate at a density of 25,000 cells/well and cultured in a myogenic differentiation medium, which was D-MEM supplemented with 2% horse serum (HS; Thermo Fisher Scientific) and 1% P/S, for 6 days to form myotubes. C2C12 myotubes were exposed to D-MEM containing 10% fetal bovine serum (FBS; Thermo Fisher Scientific) and 1% P/S with or without 100 μg mL^{-1} PSLs or 1×10^{-5} M dexamethasone (Fujifilm Wako Pure Chemical, Osaka, Japan) for 24 h. Myotubes were characterized by immunocytochemistry of myosin (a marker for myotubes). Cells were fixed with 4% paraformaldehyde/PBS, permeabilized in 0.1% Triton-X 100/PBS, and blocked with blocking buffer (PBS supplemented with 2% bovine serum albumin [Sigma–Aldrich] and 5% normal goat serum [Jackson Immuno Research Laboratories, West Grove, PA, USA]. Cells were stained with 1 μg mL^{-1} mouse anti-myosin 4 monoclonal antibody (clone: MF20) and 5 μg mL^{-1} Alexa Fluor 488-labeled goat anti-mouse IgG (H+L) highly cross-adsorbed secondary antibody (both

from Thermo Fisher Scientific) in the blocking buffer. Nuclei were stained with Hoechst 33342 (Dojindo, Kumamoto, Japan). Images were acquired and analyzed using ZOE Fluorescence Cell Imager (Bio-Rad Laboratories, Hercules, CA, USA) and ImageJ (National Institutes of Health, Bethesda, MD, USA).

Preparation of Macrophage-Conditioned Medium (CM): RAW 264.7 macrophages were grown in a 10-cm dish until they reached 70% confluency, and the medium was replaced with fresh Eagle's minimum essential medium containing 100 ng mL^{-1} lipopolysaccharide (LPS, Sigma-Aldrich) and/or 100 μg mL^{-1} PSLs. After 24 h, the medium was removed, the cells were washed twice with the medium to remove LPS and PSLs, and fresh medium was added to the dishes. After further incubation for 24 h, the medium was collected in tubes and frozen at –80 °C until further use.

Cellular Responses in Macrophage CM: For the proliferation assay, 5000 cells were seeded in 96-well plates. After 24 h of culturing, the culture medium was replaced with fresh medium containing 25% macrophage CM. On days 1 and 5, the level of viable cells was determined using Cell-Counting Kit-8 (CCK-8; Dojindo). Migration of cells against macrophage CM was examined using a Boyden chamber assay. Cells were harvested, resuspended in serum-free DMEM, and 50,000 cells were seeded into the upper 24-well chamber with a pore size of 8 μm and pore density of 1×10^5 pores cm^{-2} (Corning Inc., Corning, NY, USA). Serum-free DMEM with or without macrophage CM was added to the lower well to allow cells to migrate across the filter. After 4 and 24 h, non-migrating cells on the upper well chamber were removed using cotton swabs, while migrating cells on the underside of the filter were not treated. Then, the chambers were transferred to CCK-8 solution and incubated for 3 h at 37 °C to estimate the level of migrating cells. For the myotube formation assay, C2C12 cells were seeded in 24-well plates at an initial density of 25,000 cells/well. After culturing for 24 h, the culture medium was replaced with the myogenic differentiation medium with or without 25% macrophage CM. Negative control cells were cultured in DMEM supplemented with 10% FBS and 1% P/S. The medium was refreshed every 2 days. Myotube formation was characterized as described above, although images were acquired using an all-in-one fluorescence microscope (BZX710; Keyence) for no particular scientific reason.

Histological Analysis: For hematoxylin & eosin (HE) and Masson's trichrome (MT) staining and immunostaining, the muscles were fixed in 4% paraformaldehyde/PBS (Fujifilm Wako Pure Chemical)

and the resulting tissue was embedded in paraffin. After routine processing, the sections were stained with either HE or MT or processed for immunostaining. Immunostaining was conducted using mouse anti-rat CD68 (clone: ED1; Abcam, Cambridge, UK), mouse anti-rat CD163 (clone ED2; Bio-Rad), rabbit anti-mouse inducible nitric oxide synthase (iNOS; PA1-036, Thermo Fisher Scientific), rabbit anti-C-C-chemokine receptor 7 (CCR-7; clone: Y59, Abcam), mouse anti-paired box protein-7 (Pax-7; clone: PAX7; R&D Systems, Minneapolis, MN, USA), or rabbit anti-human-α smooth muscle actin (α-SMA; ab5694, Abcam) antibodies. Images of the stained sections were acquired using a BX43 light microscope (Olympus, Tokyo, Japan), and analyzed using CellSens imaging software (Olympus) or ImageJ.

Table S1. Pearson correlation coefficient between macrophage response and tissue responses.

	Macrophages	Capsular thickness	Number of FBGCs	Area of regenerated myofibers	Number of regenerated myofibers
1 wk	iNOS	0.34	0.27	-0.49*	-0.44
	CCR-7	0.18	0.35	-0.26	-0.19
	CD163	-0.58*	-0.52*	0.31	0.32
2 wk	iNOS	0.59*	0.67**	-0.39	-0.47*
	CCR-7	0.75*	0.54*	-0.53*	-0.58*
	CD163	-0.54*	-0.41	0.65**	0.60**

*$p < 0.05$, **$p < 0.01$.

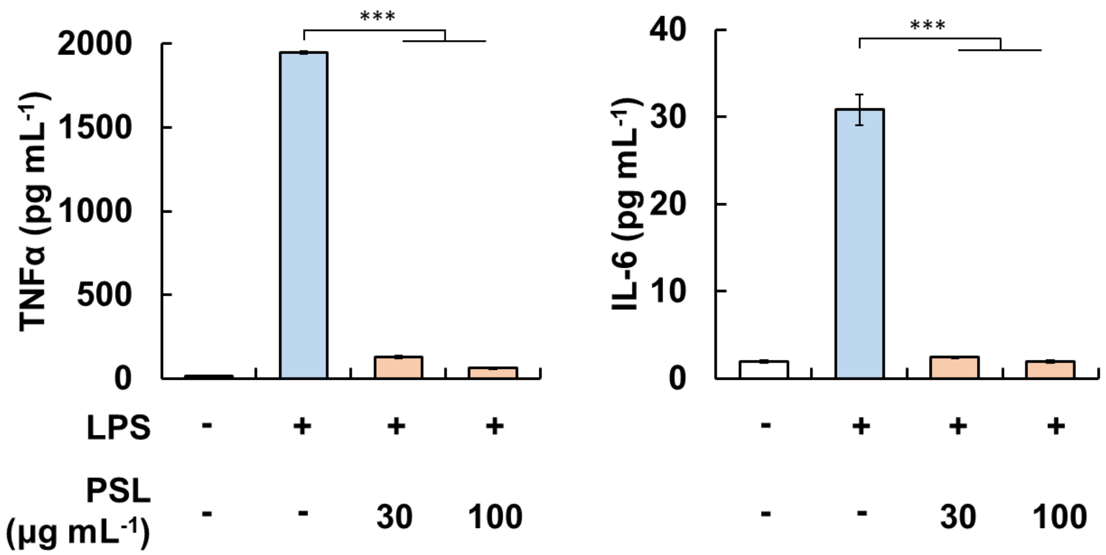

Figure S1. Cytokine profiles of M1 type human THP-1 macrophages at 24 h after treating with phosphatidylserine liposome (PSL) plus 100 ng mL^{-1} lipopolysaccharide (LPS) (n = 4). Data are presented as mean ± SD. ***$p < 0.001$.

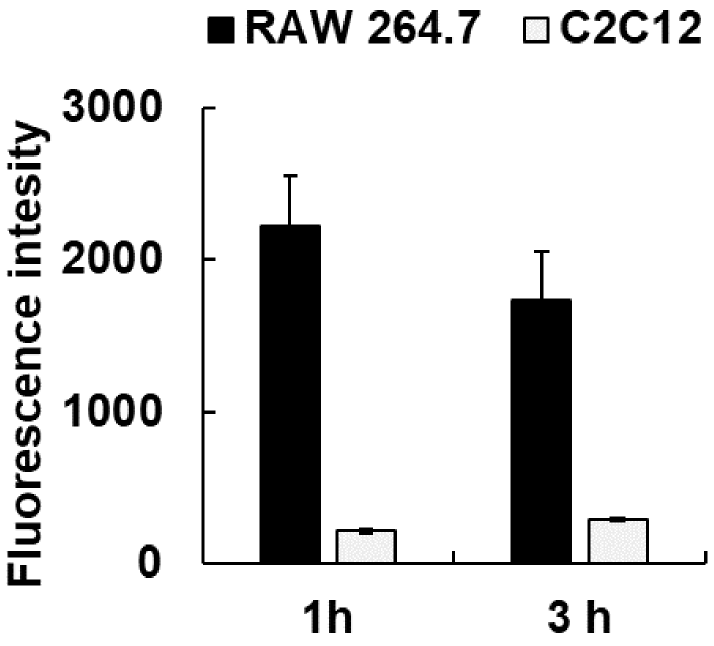

Figure S2. Cellular uptake of phosphatidylserine liposome (PSL) determined using the fluorescence intensity. Fluorescence intensity was normalized with the protein concentration in cellular lysate. Data are presented as mean ± SD (n = 4).

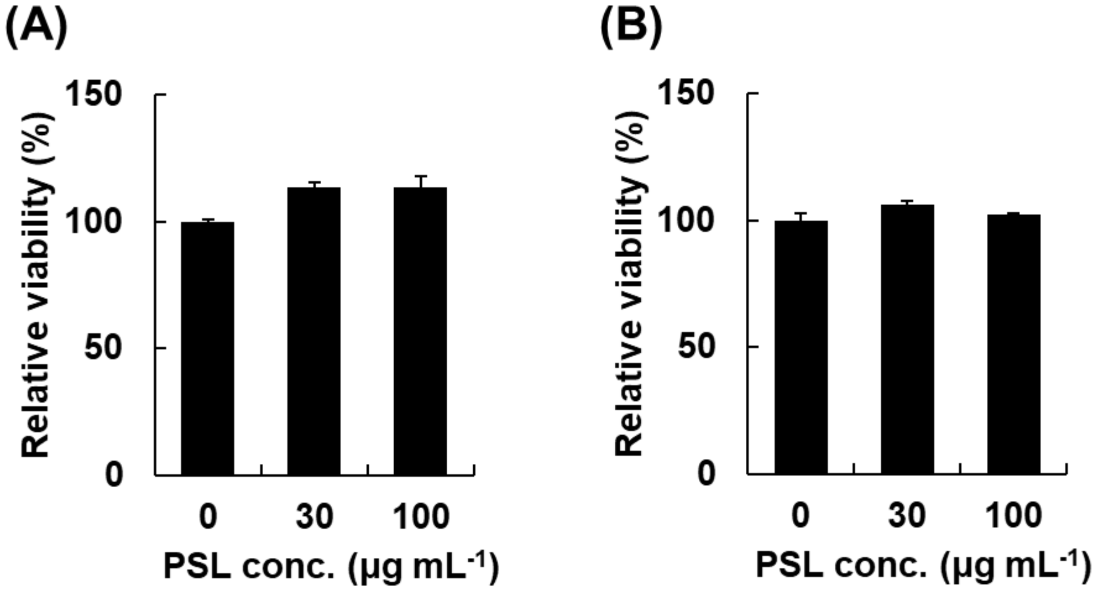

Figure S3. Phosphatidylserine liposome (PSL) is not cytotoxic toward (A) RAW 264.7 macrophages and (B) C2C12 myoblasts. Living cells were determined using Cell-Counting Kit 8. Data are presented as mean ± SD (n = 5).

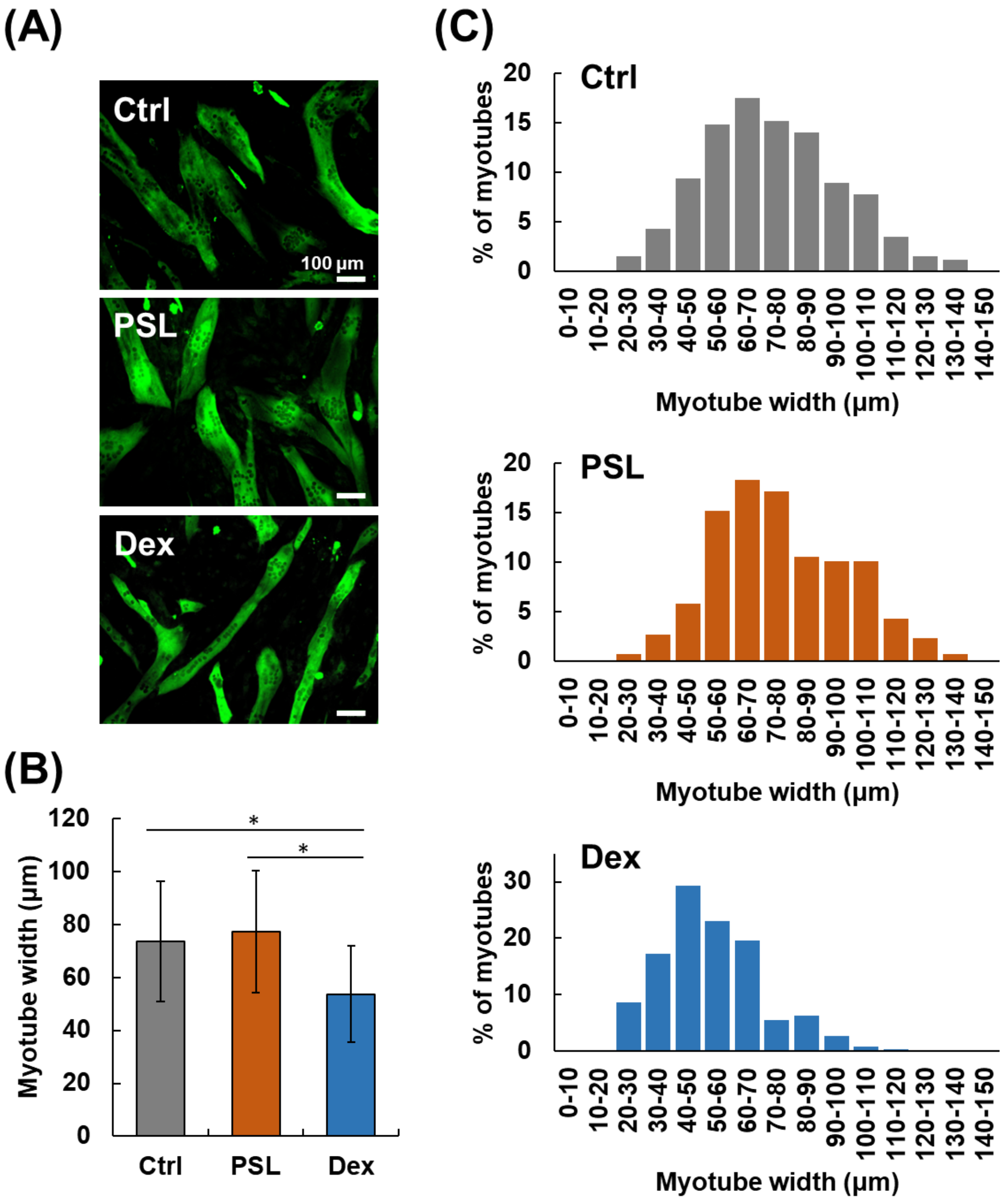

Figure S4. C2C12 myotube atrophy. (A) Images of C2C12 myotubes at 24 h after treatment with phosphatidylserine liposome (PSL) or dexamethasone (Dex). Green: Myosin heavy chain. (B) Average and (C) histogram of myotube width. Myotube atrophy was induced by Dex, not PSL. Data are presented as mean ± SD (n = 253−291). *p < 0.05.

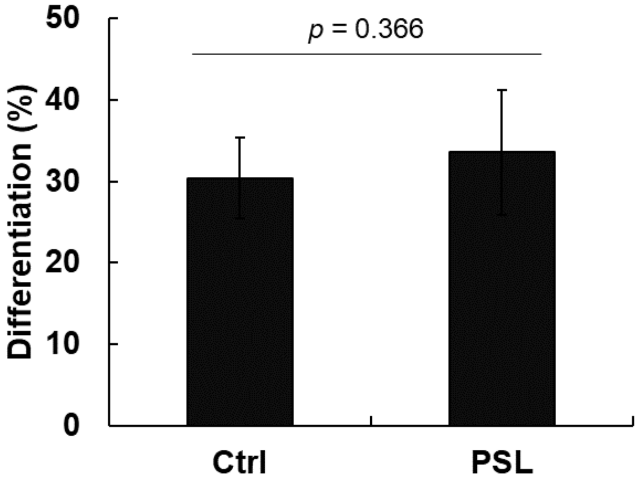

Figure S5. Phosphatidylserine liposome (PSL) has no effect on C2C12 myogenic differentiation. Control (Ctrl) is C2C12 cells cultured in myogenic differentiation medium (DMEM plus 2% horse serum) without PSL. Data are presented as mean ± SD (*n* = 4).

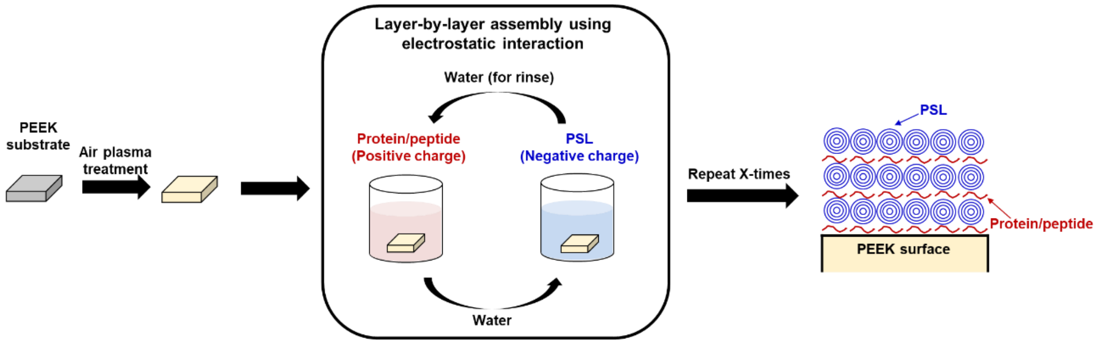

Figure S6. Schematic illustration of the phosphatidylserine liposome (PSL)-multilayer-coated poly(ether-ether-ketone) (PEEK). PSL multilayer is constructed on the PEEK surface using the layer-by-layer (LbL) method based on electrostatic interactions between polycations (i.e., protein or peptide) and negatively charged PSLs.

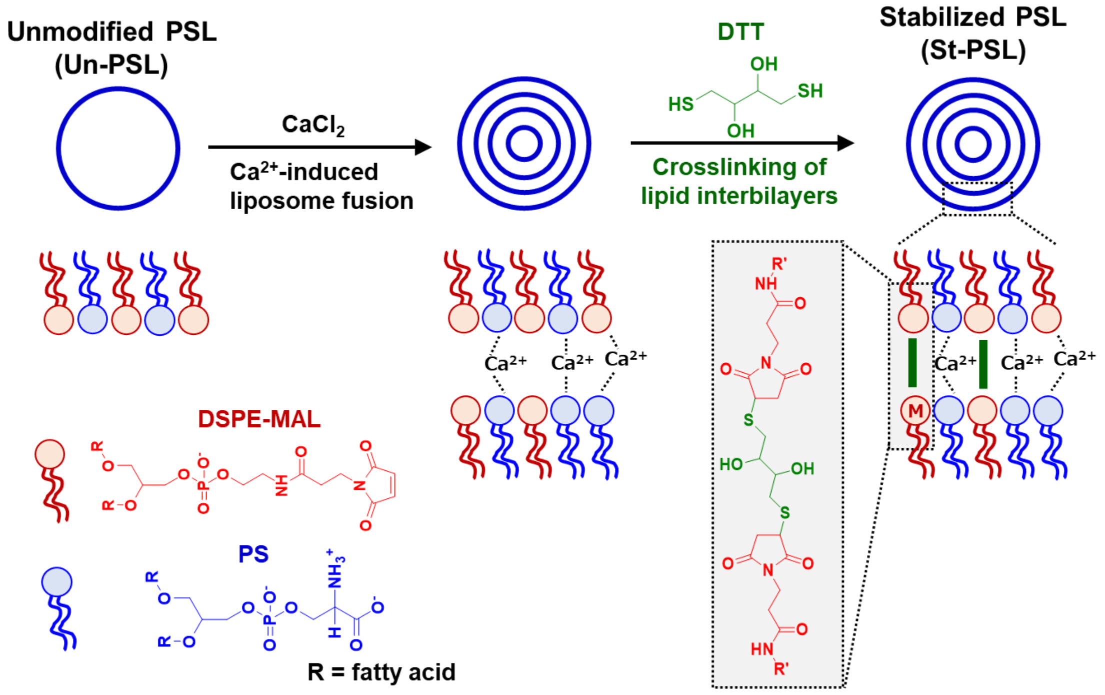

Figure S7. Scheme of a multilamellar, crosslinked interbilayer phosphatidylserine liposome. An original PSL (unmodified PSL; Un-PSL) is a unilamellar vesicle. Treatment of Un-PSL with 4×10^{-3} M $CaCl_2$ induces liposomal fusion to form the multilamellar liposome, and subsequent dithiothreitol (DTT) treatment (1 eq. to DSPE-MAL) of multilamellar liposomes crosslinks adjacent phospholipids through the Michael addition reaction between maleimide groups in DSPE-MAL and thiol groups in DTT to obtain a stabilized PSL (St-PSL).[S1]

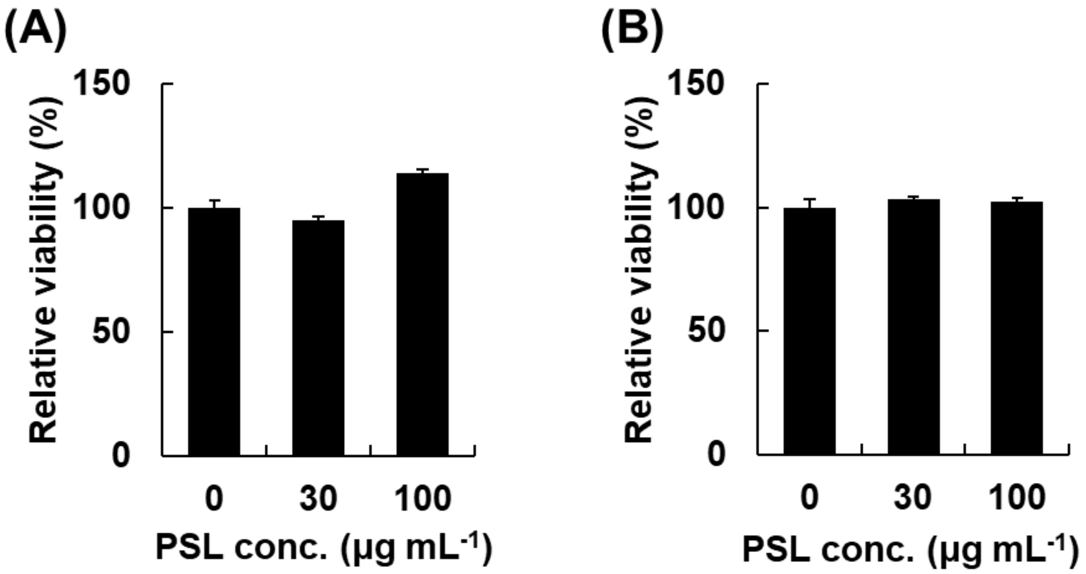

Figure S8. Multilamellar phosphatidylserine liposome (PSL) with a crosslinked structure (St-PSL) is not cytotoxic toward (A) RAW 264.7 macrophages and (B) C2C12 myoblasts. Living cells were determined using Cell-Counting Kit 8. Data are presented as mean ± SD (*n* = 3).

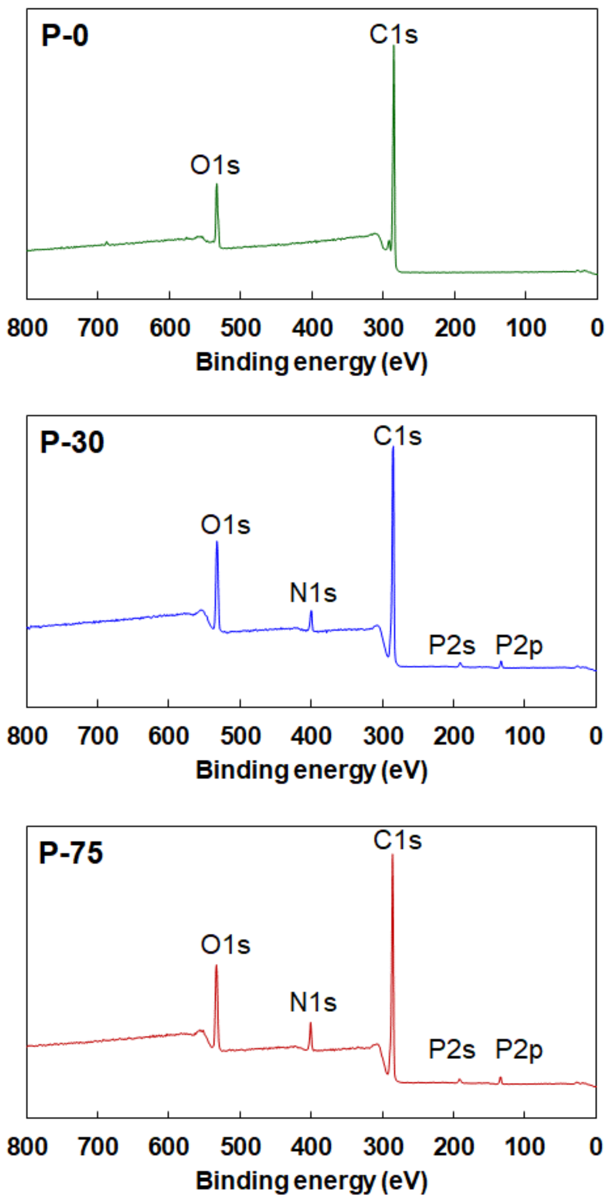

Figure S9. Wide-scan XPS spectra. Phosphatidylserine-containing liposome (PSL)-modified PEEK (P-30 and P-75) produce P2p, P2s, and N1s peaks, which are derived from phospholipids (in PSL) and protamine, indicating successful modification of PSL and protamine on PEEK.

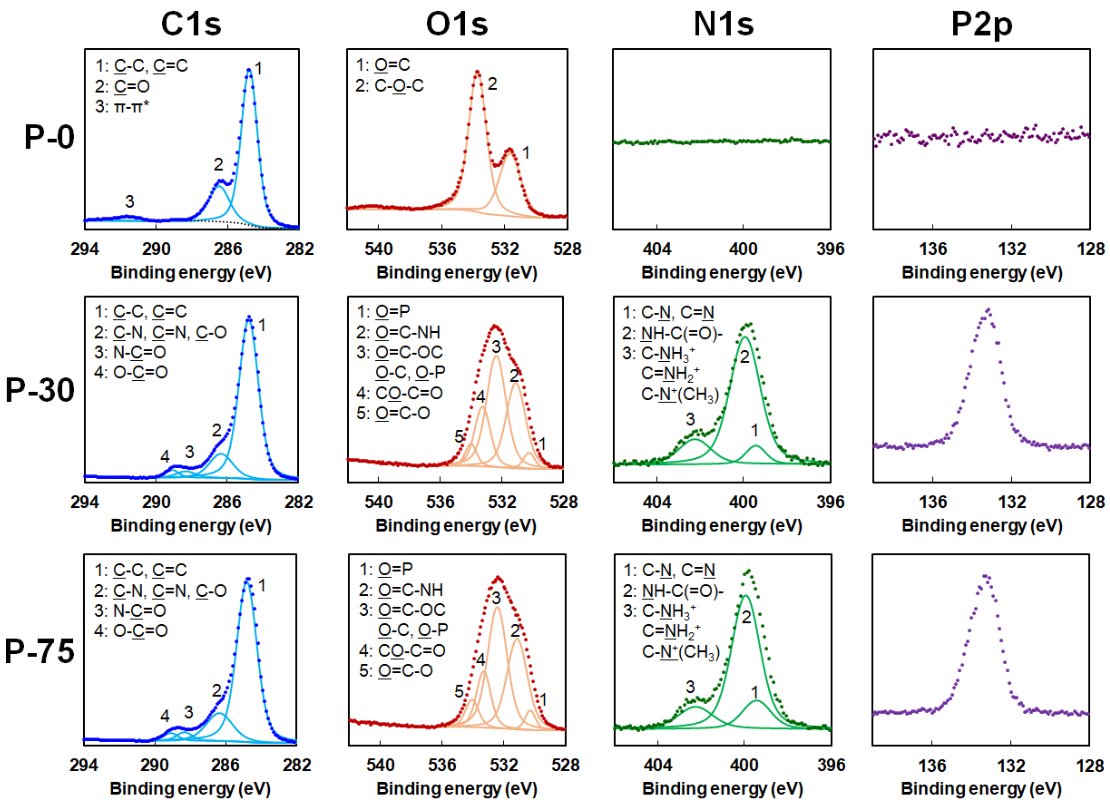

Figure S10. Core-level XPS spectra. The measured spectra are presented with dotted lines and the individual contributions of different functional groups are shown in solid lines. Phosphatidylserine-containing liposome (PSL)-modified PEEK (P-30 and P-75) exhibited similar C1s, O1s, N1s, and P2p spectra that differ from those of their original counterpart (P-0). The peaks corresponding to the functional groups of phospholipids (e.g., ester, phosphodiester, carboxylate, primary/quaternary amine, and maleimide groups) and protamine, an arginine-rich protein (e.g., amide, guanidino, alcohol, carboxylate, and primary amine groups), were identified in P-30 and P-75, but not in P-0. The absence of ketone and π–π* peaks (both originating from the PEEK substrate) in P-30 and P-75 also confirmed that PSL multilayer completely covered the PEEK surface. Please see Figure S11 for chemical structures of PEEK and phospholipids used.

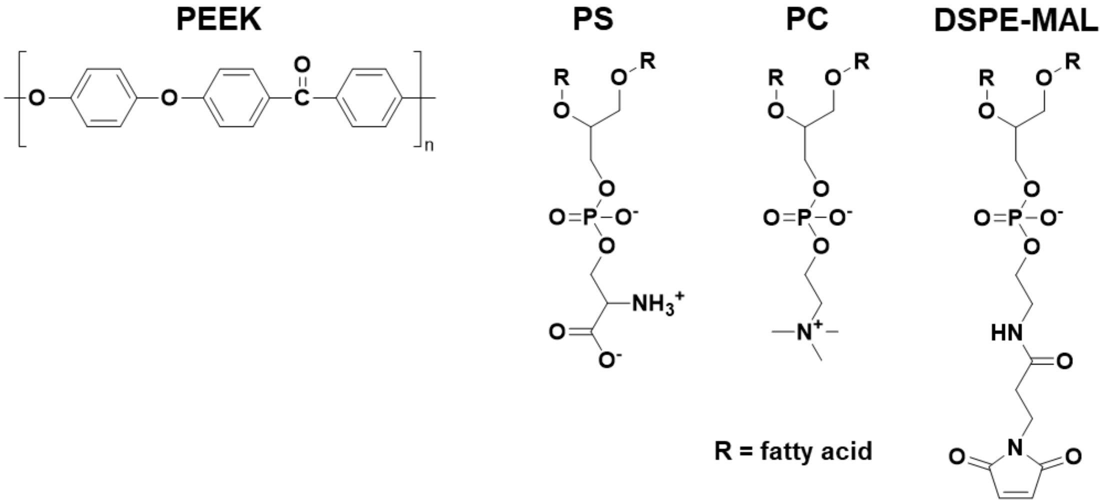

Figure S11. Chemical structures of poly(ether-ether-ketone) (PEEK), phosphatidylserine (PS), phosphatidylcholine (PC), and 1,2-distearoyl-*sn*-glycero-3-phosphoethanolamine-maleimide (DSPE-MAL).

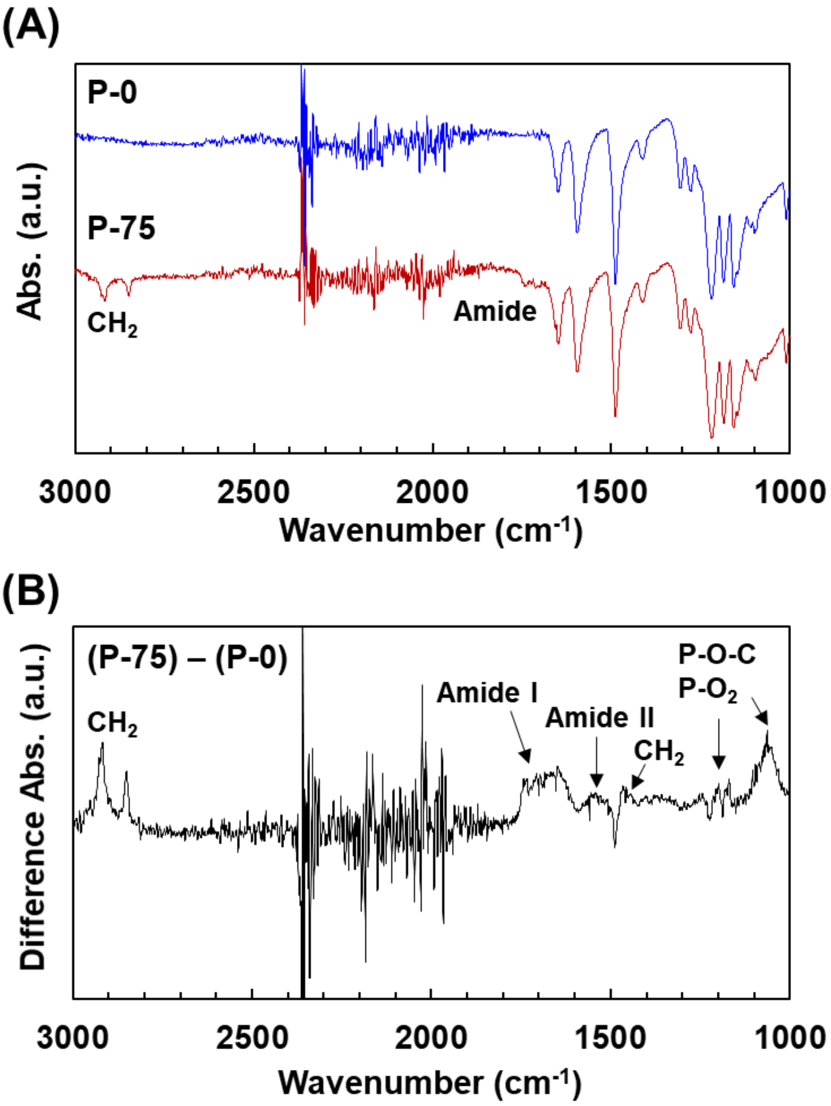

Figure S12. (A) Attenuated total reflection-Fourier transform infrared (ATR-FTIR) spectra of bare (P-0) and liposome-coated PEEK (P-75). (B) ATR-FTIR difference spectra of P-75 subtracted by P-0. Please see Figure S11 for chemical structures of PEEK and phospholipids used.

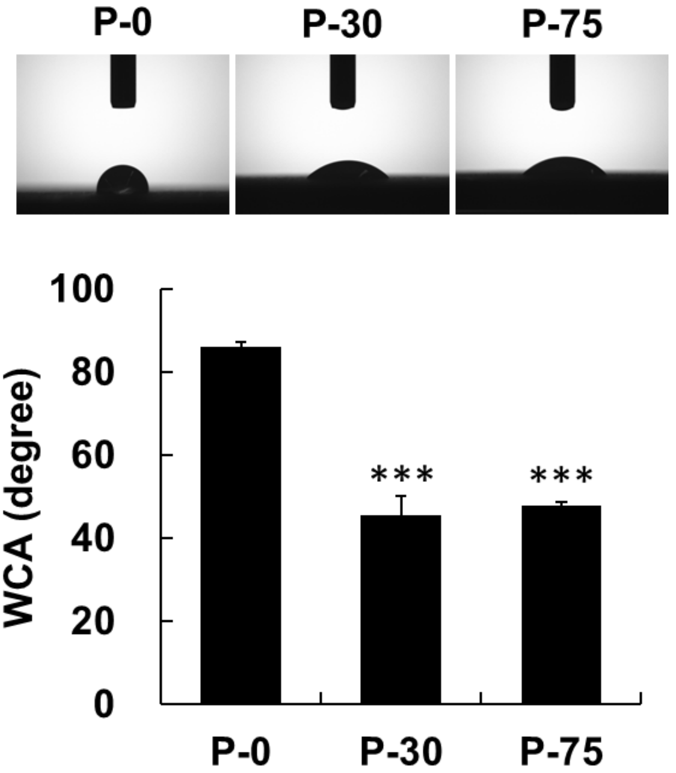

Figure S13. Water contact angle (WCA) of samples (n = 3). P-0, original PEEK; P-30 and P-75, PEEK coated using layer-by-layer assembly for 30 and 75 cycles, respectively. Data are presented as mean ± SD. ***$p < 0.001$ (vs. P-0).

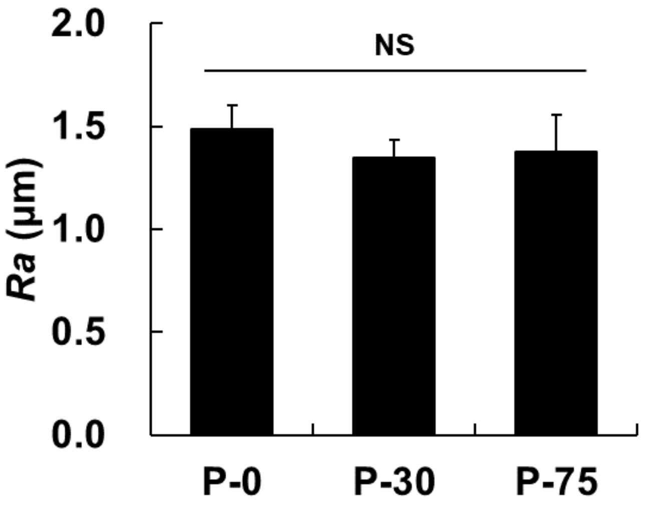

Figure S14. Average surface roughness of samples (*n* = 3). P-0, original PEEK; P-30 and P-75, PEEK coated using layer-by-layer assembly for 30 and 75 cycles, respectively. Data are presented as mean ± SD. NS, not significant.

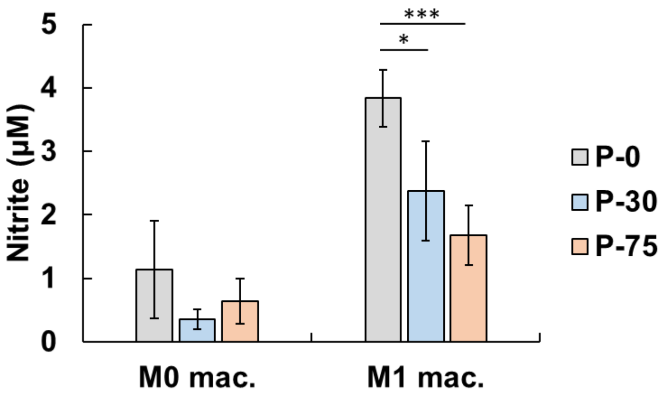

Figure S15. Nitric oxide (NO) production from M0 and M1 macrophages cultured on samples. The stable metabolite of NO, nitrite, was measured using the Griess–Romijn nitrite reagent.[S1] P-0, original PEEK; P-30 and P-75, PEEK coated using layer-by-layer assembly for 30 and 75 cycles, respectively. Data are presented as mean ± SD (n = 5). *$p < 0.05$, ***$p < 0.001$.

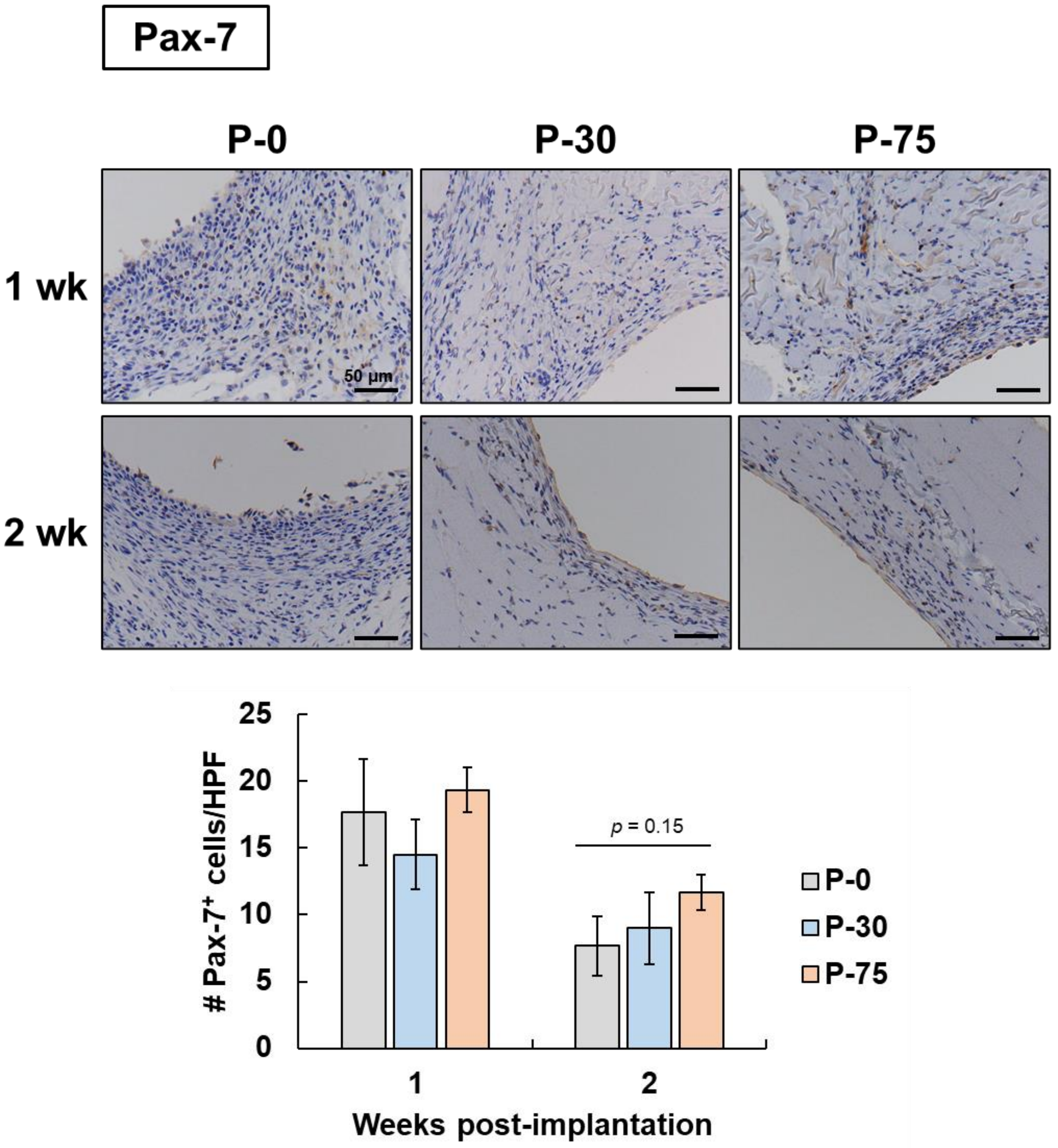

Figure S16. Phosphatidylserine liposomes (PSLs) multilayer had no effect on skeletal muscle satellite cell recruitment at the injured or repaired muscle. Paired box 7 protein (Pax-7) is used as a marker of skeletal muscle satellite cells, and positive cells per high power filed (HPF; original magnification ×400) were counted (n = 6). P-0, original poly(ether-ether-ketone) (PEEK); P-30 and P-75, PEEK coated using layer-by-layer assembly for 30 and 75 cycles, respectively. Scale bar = 50 μm. Data are presented as mean ± SD.

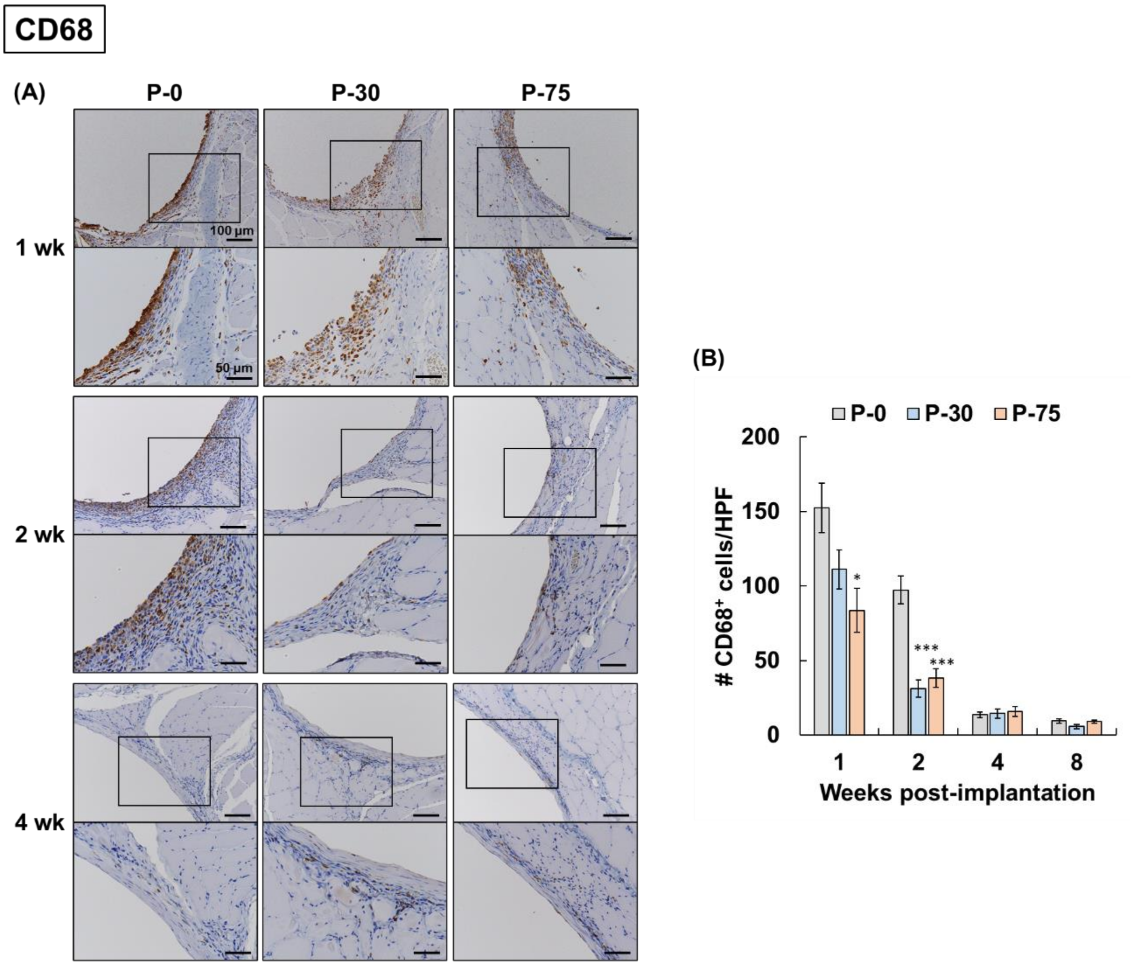

Figure S17. Macrophage infiltration surrounding liposome multilayer-coated poly(ether-ether-ketone) (PEEK) rods. (A) Representative images of CD68 (a pan-macrophage marker)-stained section at 1−4 weeks post-implantation (original magnification 200× [upper images] and 400× [lower images]). Scale bars = 100 μm (upper images) and 50 μm (lower images). P-0, original PEEK; P-30 and P-75, PEEK coated using layer-by-layer assembly for 30 and 75 cycles, respectively. (B) Number of $CD68^+$ cells per high power field (HPF; original magnification 400×) (n = 6). Data are presented as mean ± SEM. *$p < 0.05$; ***$p < 0.001$ (vs. P-0).

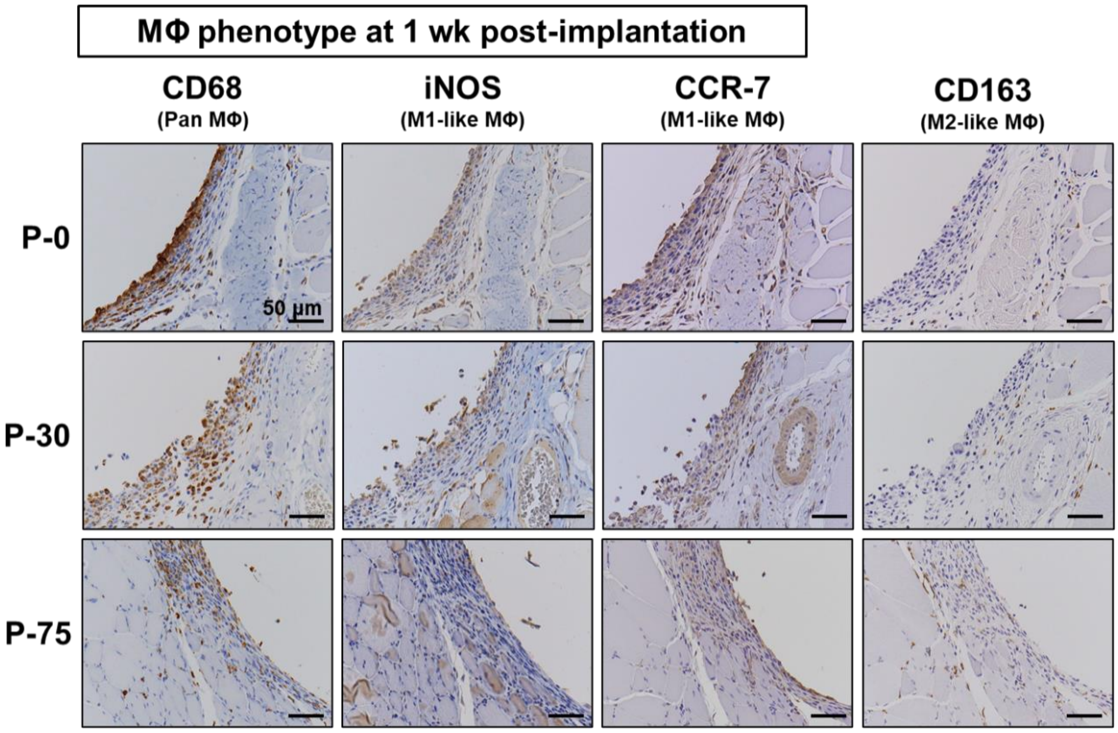

Figure S18. Representative images of inducible nitric oxide synthase (iNOS), C-C chemokine receptor type 7 (CCR-7), and CD163-stained histological sections at 1 week post-implantation (original magnification 400×). Scale bars = 50 μm. iNOS and CCR-7 are markers for M1 macrophages, and CD163 is a marker for M2 macrophages. P-0, original PEEK; P-30 and P-75, PEEK conducted a layer-by-layer assembly process for 30 and 75 cycles, respectively. Note that the same images of CD68-stained sections as seen in Figure S17 are provided to easily understand the macrophage distribution.

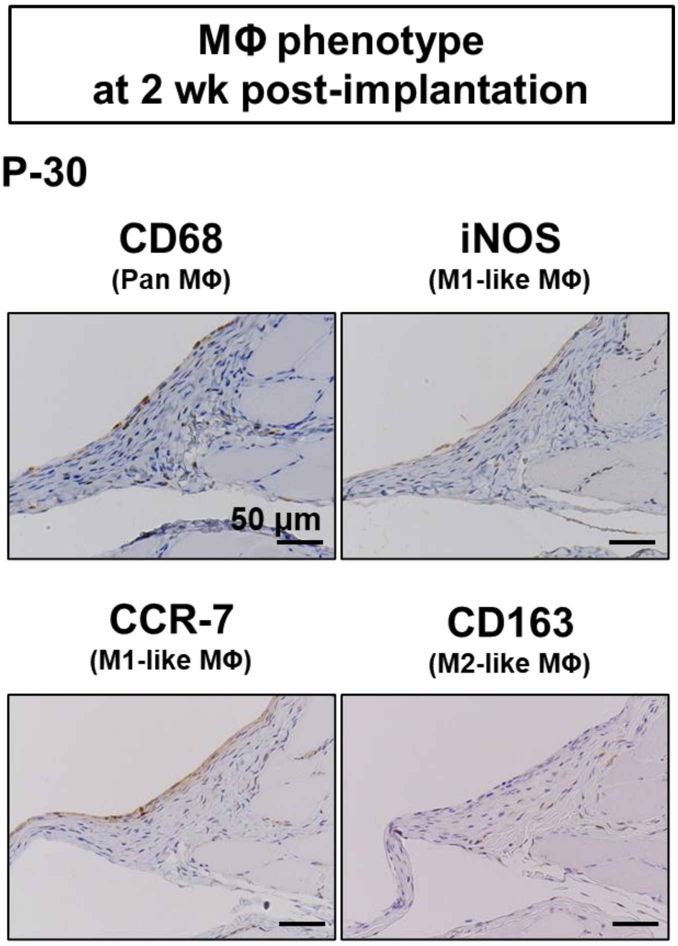

Figure S19. Representative images of inducible nitric oxide synthase (iNOS), C-C chemokine receptor type 7 (CCR-7), and CD163-stained histological sections at 2 weeks post-implantation (original magnification 400×). Scale bars = 50 μm. P-30, PEEK coated using layer-by-layer assembly for 30 cycles. Note that the same images of CD68-stained sections shown in Figure S17 are provided to easily understand the macrophage distribution.

References

[S1]R. Toita, J. H. Kang, A. Tsuchiya, *Acta Biomater.* **2022**, *154*, 583.

[S2]L. Wang, X. F. Jiao, C. Wu, X. Q. Li, H. X. Sun, X. Y. Shen, K. Z. Zhang, C. Zhao, L. Liu, M. Wang, Y. L. Bu, J. W. Li, F. Xu, C. L. Chang, X. Lu, W. Gao, *Cell Death Discov.* **2021**, *7*, 251.
